# Exploring the Nature of *Arhopalus ferus* (Coleoptera: Cerambycidae: Spondylidinae) Pheromone Attraction

**DOI:** 10.1007/s10886-024-01508-8

**Published:** 2024-06-06

**Authors:** Jessica L. Kerr, Cecilia M. Romo, Brooke O’Connor, Georgia Dickson, Max Novoselov, Samuel Aguilar-Arguello, Christine Todoroki, Adriana Najar-Rodriguez, Lee-Anne Manning, Andrew Twidle, Anne Barrington, Gaetan Leclair, Peter Mayo, Jon Sweeney

**Affiliations:** 1 Scion (New Zealand Forest Research Institute Limited), 10 Kyle Street, Riccarton, Christchurch, 8011 New Zealand; 2 Scion (New Zealand Forest Research Limited), Te Papa Tipu Innovation Park, Tikokorangi Drive, Rotorua, New Zealand; 3 Plant and Food Research, Canterbury Agriculture & Science Centre, 74 Gerald St, Lincoln, 7608 New Zealand; 4 Plant and Food Research, 120 Mt Albert Road, Sandringham, Auckland, 1025 New Zealand; 5 Natural Resources Canada - Canadian Forest Service, Atlantic Forestry Centre, 1350 Regent Street, Fredericton, New Brunswick E3C 2G6 Canada

**Keywords:** Cerambycidae, Aggregation-sex pheromone, Fuscumol, Geranylacetone, Spondylidinae

## Abstract

Cerambycid species of the Spondylidinae subfamily are distributed worldwide and are known for being prolific invaders that infest conifers. In New Zealand, *Arhopalus ferus* (Mulsant), the burnt pine longhorn beetle, is well-established and requires monitoring at high-risk sites such as ports, airports, and sawmills as part of the requirements to meet pine log export standards set by the New Zealand Ministry of Primary Industries (MPI). Currently, its surveillance relies on traps baited with host volatiles (i.e., ethanol and α-pinene). We used volatile collections from adult beetles, electroantennograms, and field trapping bioassays to identify the pheromones emitted by the burnt pine longhorn beetle *A. ferus* and their effects on its behaviour. We show that *A. ferus* males emit mainly (*E*)-fuscumol and geranylacetone, as well as the minor components, *α*-terpinene and *p*-mentha-1,3,8-triene, and that all four compounds elicit a dose-dependent response in antennae of both sexes. Traps baited with the binary combination of geranylacetone plus fuscumol captured significantly more female *A. ferus* than did unbaited traps in two of three field experiments. α-Terpinene did not affect *A. ferus* trap catches and effects of *p*-mentha-1,3,8-triene on trap catch were not determined. Our findings provide further evidence of the use of fuscumol and geranylacetone as aggregation-sex pheromones by longhorn beetles in the Spondylidinae subfamily, and suggest that their deployment in survey traps may improve the efficacy of *A. ferus* monitoring in New Zealand and elsewhere.

**Supplementary Information:**

The online version contains supplementary material available at 10.1007/s10886-024-01508-8.

## Introduction

Longhorned beetles (Coleoptera: Cerambycidae) are among the most common phytophagous pests spread globally within wood and wooden products and are among the beetles most likely to be intercepted in international quarantine (Brockerhoff et al. 2006; Eyre and Haack 2017; Haack 2017). Increased awareness of the economic and ecological importance of cerambycid beetles has led to a dramatic increase in the study of their chemical ecology with more than 300 adult aggregation-sex pheromones identified in the past two decades (Allison et al. 2004; Hanks and Millar 2016), several of which have been synthesized and used for baits in trapping surveys and monitoring programs. There is increasing evidence that many cerambycid pheromone structures are broadly shared among congeners and species in different tribes and subfamilies in different parts of the world (Boone et al. 2019; Hanks and Millar 2013; Millar et al. 2017; Mitchell et al. 2011). To date, the pheromones identified from species in the subfamilies Cerambycinae, Lamiinae, and Spondylidinae are all male-produced aggregation-sex pheromones attracting both sexes whereas those emitted by species in the Prioninae and Lepturinae are female-produced sex pheromones attracting only males (Millar and Hanks 2017).

Of the known male-produced aggregation-sex pheromones common among the Cerambycidae, fuscumol ((*E*)-6,10-dimethyl-5,9-undecadien-2-ol), fuscumol acetate ((*E*)-6,10-dimethyl-5,9-undecadien-2-yl acetate) and geranylacetone ((*E*)-6,10-dimethyl-5,9-undecadien-2-one) are found in many species of Spondylidinae and Lamiinae. Both fuscumol and fuscumol acetate are chiral compounds and depending on the species, the presence of both (*R*)*-* and (*S*)*-* enantiomers of fuscumol or fuscumol acetate may synergize or inhibit attraction (Hughes et al. 2016; Meier et al. 2016, 2020). For example, male *Astylidius parvus* (LeConte)(Lamiinae) produce (*R*)- and (*S*)-fuscumol, (*R*)-fuscumol acetate, and geranylacetone, whereas males of *Lepturges angulatus* (LeConte) produce (*R*)- and (*S*)-fuscumol acetate and geranylacetone; adult beetles were attracted to only their species-specific blend of enantiomers of fuscumol or fuscumol acetate, respectively, and not to individual enantiomers (Meier et al. 2016). Another terpenoid compound, (*S*)-6-methylhept-5-en-2-ol ((*S*)*-*sulcatol) was also reported as the aggregation-sex pheromone for the lamiines *Astylopsis macula* (Say) and *Leptostylus transversus* (Gyllenhal) (Meier et al. 2019).

In the Spondylidinae subfamily, (*S*)-fuscumol and geranylacetone are emitted as major and minor components, respectively, by *Arhopalus rusticus* (Linnaeus) (Žunič-Kosi et al. 2019) and *Arhopalus productus* (LeConte) (Hanks and Millar 2016). *Arhopalus rusticus* were attracted by (*S*)-fuscumol and its racemate but the former was more attractive. (*S*)*-*Fuscumol is also emitted by *Tetropium fuscum* (Fabricius) and *T. cinnamopterum* (Kirby), and both (*S*)*-* and racemic fuscumol synergize attraction of these species as well as their congener *T. castaneum* (Linnaeus) to host plant volatiles (Silk et al. 2007; Sweeney et al. 2010). Racemic fuscumol also attracts *Tetropium abietis* (Halloran et al. 2018), *T. gabrieli* (Weise) (Schroeder et al. 2021), and the combination of (*E/Z*)*-*fuscumol and (*E/Z*)*-*fuscumol acetate attracts *T. schwarzianum* (Casey) (Hanks and Millar 2013). *Asemum nitidum* (LeConte) produces and responds to the combination of (*S*)-fuscumol and geranylacetone whereas the congeneric *A. caseyi* (Linsley) produces and responds to geranylacetone alone (Halloran et al. 2018).

Members of the Spondylidinae subfamily are distributed worldwide but most are native to the Northern hemisphere where they are sporadically recorded infesting conifers, such as larch, fir, and pine. A few species, notably in the genus *Arhopalus*, have become prolific invaders, successfully establishing in conifer plantations in New Zealand, Australia, South Africa, and South America (Halloran et al. 2018; Lynikienė et al. 2021; Žunič-Kosi et al. 2019). *Arhopalus rusticus*, invasive in Australia and South America (Fachinetti and Grilli 2020; Wang and Leschen 2003), is known to carry larvae of *Bursaphelenchus xylophilus* (Wang and Leschen 2003), the phytopathogenic nematode that causes pine wilt disease which threatens pine forests worldwide (Linit 1988). *Arhopalus syriacus* (Reitters) is invasive in Australia, South Africa and South America (Brockerhoff 2009; Fachinetti and Grilli 2020; Wang and Leschen 2003). Another species of Palearctic origin, *A. ferus*, the burnt pine longhorn beetle, is invasive in New Zealand (Brockerhoff et al. 2006) and South Africa (Grobbelaar 2017).

In New Zealand, *A. ferus* is one of the most common forest insects in pine plantations (Pawson et al. 2020a), fire wood yards, and sawmills and is regularly monitored as a quarantine pest at ports where pine logs are exported (Brockerhoff et al. 2006; Pawson et al. 2020b). Originally from Europe, *A. ferus* was first detected in New Zealand in 1963. Its larvae develop in stumps, large branches, and can occasionally infest and kill stressed or fire-damaged *Pinus radiata* (D. Don). *Arhopalus ferus* is attracted to the volatiles emitted from freshly cut logs and fire-damaged pines (Hosking 1978). Current surveillance methods and monitoring of this species are limited to host volatile lures (ethanol and *α*-pinene) baited on black panel flight interception traps which have proven effective in Europe (Jurc et al. 2012) as well as New Zealand (Brockerhoff et al. 2006). However, recent tests in New Zealand found that *A. ferus* catch in traps baited with ethanol and *α*-pinene was no different from that in unbaited traps (Kerr et al. 2022). This contradictory result in New Zealand raised questions as to whether a more species-specific lure could be developed for monitoring *A. ferus*. With the recent detection of pheromones of its congener, *A. rusticus* (Žunič-Kosi et al. 2019), studies to investigate pheromones of *A. ferus* and their potential to improve its surveillance were initiated. Kerr et al. (2022) found that racemic fuscumol increased trap catch of *A. ferus* by 50% compared to unbaited traps but the effect was only marginally significant and suggested further investigation was warranted.

Thus, the aims of this study were to: (1) collect and analyse effluvia from male and female *A. ferus* and determine structures of putative pheromone compounds present; (2) test whether these compounds induced antennal response in *A. ferus*; and (3) field test pheromone candidates for their effects on catch of *A. ferus* in flight intercept traps.

## Methods and Materials

### Headspace Gas Volatile Collection

*Arhopalus ferus* adults were hand-collected from freshly cut *P. radiata* logs at SRS (Shands Road Sawmills Ltd.) sawmill (Rolleston, Canterbury) over several evenings during March–April 2021. Beetles were sexed the same evening they were collected (by eye based on body size and shape of the terminal abdominal tergite) and stored at 15 °C in plastic containers loaded with sugar water and damp towels for hydration, inside an incubator (MIR-153 Sanyo, Osaka, Japan). Prior to use in volatile collections, the sex of individuals was confirmed under a stereomicroscope at 10X magnification. Effluvia samples were collected from six groups (i.e., replicates) of five males, and six groups of five females each collected on different nights, and two controls (clean, empty glass chambers) during 4 weeks in March and April 2021. Individual beetles were placed in a clean glass chamber (250 × 45 × 35 mm) lined with half a piece of filter paper (90 mm diameter, Whatman plc, United Kingdom) (Supplementary Fig S1) to provide the beetle with greater traction and minimise the risk of the beetle getting stuck on its back and struggling to right itself. Volatiles were collected in glass tube columns (0.635 × 17.8 cm, Sigma Aldrich, United States) containing 150 ± 5 mg of HayeSep® Q adsorbent (80/100 mesh, Sigma Aldrich) and plugged with glass wool (Silanized, Sigma Aldrich) on both ends. Air was pulled first through an activated charcoal filter (Restek, United States), then through the glass chamber containing the beetles, and finally through the adsorbent column at a rate of 0.25 Lmin^− 1^. Volatiles were collected from each group of beetles for 24 h, under natural lighting conditions approximate L: D 11.5:12.5, with the laboratory held at 18 °C. Two controls, consisting of identical clean glass chambers containing filter paper but no beetles, were sampled similarly. Volatiles were eluted from the glass columns with four 160-µl aliquots of dichloromethane (99%, Sigma Aldrich) into amber glass vials (Thermofisher Scientific, United States), which were then stored at -18 °C for chemical analyses. Glassware was cleaned by soaking in a 5% Decon 90 solution (Decon Laboratories Ltd, United Kingdom) for at least 20 min and rinsed with deionized water and air dried (in a fume hood) between each replicate. The charcoal filter was replaced weekly over the 4 weeks of the experiment.

### Pheromone Sources and Lures

Racemic (*E*)-fuscumol and (*E/Z*)-geranylacetone (60% *E*, 40% *Z*) were purchased from Bedoukian (Danbury, CT, USA), 4’-ethyl acetophenone and *α*-terpinene were purchased from Sigma-Aldrich (Oakville, ON, Canada), and *p*-mentha-1,3,8-triene (PMT) was purified (to 54%) from Parsley essential oil (Lotus Oils, Waipukurau, New Zealand) using medium-pressure column chromatography on 230–400 mesh silica gel (Sigma-Aldrich), eluting with a hexane/ethyl acetate gradient solvent system. Lures of (*E/Z*)-fuscumol and (*E/Z*)-fuscumol acetate (60% *E*, 40% Z) were purchased from Andermatt Canada (Fredericton, NB, Canada). (*E/Z*)-Fuscumol used in electrophysiological responses trials was synthesized at Natural Resources Canada, Atlantic Forestry Centre (NRCan, AFC) by lithium aluminium hydride reduction of (*E/Z*)-geranylacetone. (*R*)*-*(*E*)-Fuscumol, and (*S*)*-*(*E*)-fuscumol were synthesized by enzymatic chiral resolution of racemic (*E*)-fuscumol (Sweeney et al. 2010). (*E*)-geranylacetone was synthesized by pyridinium chlorochromate (PCC) oxidation of (*E*)-fuscumol buffered with sodium acetate in dichloromethane (Corey and Suggs 1975) at NRCan, AFC. For field experiments, each pheromone was loaded onto separate rubber septa (Natural Resources Canada, Atlantic Forestry Centre, NB, Canada), except for *α*-terpinene which was loaded into 2 gram pre-slit septa screw cap GC-MS amber glass vials (Agilent Technologies, Santa Clara, CA, USA). Percent purity, load (in mg) per septum and average release rate for each lure are reported in Table S1 (Supplementary data).

### *Arhopalus ferus* Antennal Responses to a Range of Doses of Pheromone Compounds

The electrophysiological responses of male and female *A. ferus* antennae to components identified in the headspace-gas volatile collections were recorded using electroantennography (EAG). Recordings were conducted in 2021 using laboratory-reared individuals sourced from a New Zealand insect rearing facility (Plant and Food Research, Mt Albert, Auckland, New Zealand). Larvae were reared on artificial diet for six weeks, following methods described by Barrington et al. (2015). They were then transferred to individual 60 ml plastic cups (each punctured with two ventilation holes) and fed *ad libitum* with artificial diet. The larvae were reared under these conditions at 20 °C L: D 16:8 in controlled temperature rooms for 12 weeks, then chilled (10 °C L: D 0:24) for 8 weeks to synchronise pupation. Newly emerged adults were then shipped to Plant and Food Research, Lincoln campus and held in rearing cups at 18 °C L: D 16:8 cycle until they reached sexual maturity (i.e., between 10 and 14 days old, according to (Barrington and Logan 2015) prior to EAG recordings.

We measured antennal responses to racemic (*E/Z*)-fuscumol, (*R*)*-*(*E*)-fuscumol, (*S*)*-*(*E*)-fuscumol, (*E/Z*)-geranylacetone, 4’-ethyl acetophenone, *α*-terpinene, and *p*-mentha-1,3,8-triene (PMT). All compounds were diluted with n-hexane (VWR, BDH Chemicals, 99%) then applied at 0.1, 1, 10, 100 µg doses to pieces of filter paper (1.5 × 1 cm) and exposed to air for 30 s to allow the n-hexane to evaporate. Each filter paper was then placed into an individual glass Pasteur pipette (150 mm; Volac, United Kingdom) which served as the stimulus cartridge. Each cartridge was used twice before being discarded and a new one made. The stimulus puff was applied to insects for 0.3 s via a stimulus flow controller (CS-05, Syntech, NL). The continuous air flow was charcoal-filtered and humidified and was set at 600 cm^3^/min. Antennal responses were recorded by a preamplifier (IDAC 4, Syntech, NL) and captured with EAGPro software (Syntech, Hilversum, Netherlands).

All manipulations were conducted using a stereomicroscope (Leica MZ12; objective lens Planapo 1.0 x, ocular lens 10 x). Signal electrodes consisted of silver wire (Sigma-Aldrich, 99.9%, 4.3 mm x 0.5 mm OD.) inserted into a tapered glass capillary (Drummond, USA, 25 mm x 1 mm ID.) filled with ringer’s solution (Kaissling 1995), which was screwed into an electrode holder connected to the recording and reference electrodes. Antennae were excised using a micro-blade (FST, Canada) and the exposed ends were connected to the signal electrodes with ringers’ solution. Each antennal preparation was exposed to each of the test compounds in random order. However, all four doses of each compound were tested in succession, starting with the lowest (0.1 ug) and ending with the highest (100 ug) dose, before randomly selecting another compound to test. A stimulus puff of air containing the control (1 µg of 99% n-hexane), followed by a stimulus puff of the positive control *α*-pinene (1 µg), was applied at the beginning and end of each pheromone compound tested (hereafter ‘block’). The *α*-pinene puff was simply a qualitative check to ensure the antennal preparation was still responsive and was not used in any quantitative way in the data analyses. The antennal preparation was given 1 min to recover between each stimulus puff of control stimulus or pheromone dose.

### Field Trapping Experiments

We conducted three field trapping experiments between February 2021 and March 2023 to determine the effects of various pheromone candidates (including those detected from the adult beetle effluvia) on catches of *A. ferus*. All experiments used black panel flight intercept traps (described in detail in Kerr et al. (2017)). The panels and the dry collection cups were sprayed with a contact insecticide (RipCord Plus, BASF New Zealand Limited, Auckland, New Zealand) to kill and preserve *A. ferus* that landed on or in the traps. For the second (2022) and third experiment (2023), the trap panels were sprayed with a PTFE aerosol surface lubricant (ROCOL®, Leeds, UK) to increase capture efficiency by increasing slip (Allison et al. 2011; Kerr et al. 2017). All tests of pheromone candidates were replicated in randomized complete blocks with 20 m spacing between traps, 30 m spacing between blocks and a 20 m buffer from the edge of the field site. Traps were cleared weekly, and samples stored in a -18 °C freezer until processed to record the numbers and sex of *A. ferus* collected.

#### Experiment 1: Effect of Racemic Fuscumol, Fuscumol Acetate, and Geranylacetone on Trap Catch

This experiment was conducted prior to our analysis of *A. ferus* effluvia, and was based on the positive response of *A. ferus* to racemic fuscumol in previous field trials (Kerr et al. 2022), the use of fuscumol and geranylacetone by *A. rusticus* (Žunič-Kosi et al. 2019), and the presence of fuscumol acetate in pheromones of other longhorned beetles (Meier et al. 2016). We compared responses of *A. ferus* to racemic (*E/Z*)*-*fuscumol (Ra), racemic (*E/Z*)*-*fuscumol acetate (FA) and (*E/Z*)*-*geranylacetone (GA) alone and in all combinations. There were eight different lure treatments: (1) Ra; (2) FA; (3) GA; (4) Ra + FA; (5) Ra + GA; (6) FA + GA; (7) Ra + FA + GA; and (8) control (unbaited trap). The field experiment was conducted in a *P. radiata* plantation located at Chaney’s Forest (43°25’19” S; 172°40’43” E, Rayonier Matariki Forests, Christchurch, New Zealand), from February 9 to March 16, 2021. The site had been logged and re-established 4–6 months prior to the start of the 5-week experiment. Each of the eight treatments was replicated eight times in randomized complete blocks with traps placed in an 8 × 8 formation grid (64 total traps). All lures were replaced after 3 weeks.

#### Experiment 2: Effect of Fuscumol Chirality and Geranylacetone on Trap Catch

In the second experiment, we tested the effect of fuscumol, its chirality [by comparing (*R*)-(*E*)-fuscumol (R); racemic (*E*)-fuscumol (Ra); and (*S*)-(*E*)-fuscumol (S)] and the effect of adding (*E*)-geranylacetone (GA). There were eight pheromone treatments: (1) (*S*)-fuscumol; (2) (*R*)-fuscumol; (3) racemic (*E*)-fuscumol; (4) (*E*)-geranylacetone; (5) (*S*)-fuscumol + (*E*)-geranylacetone; (6) (*R*)-fuscumol + (*E*)-geranylacetone; (7) racemic (*E*)-fuscumol + (*E*)-geranylacetone; and (8) unbaited control. The experiment was conducted in a *P. radiata* plantation forest located in Bottle Lake Forest Park (43°26’43”S, 172°41’57”E, Rayonier Matariki Forests, Christchurch, New Zealand) in a 6-month-old cutover from January 13 to April 7, 2022. Treatments were replicated 10 times in randomized complete blocks with traps deployed in a 10 × 8 formation grid (80 total traps). All lures were replaced every 4 weeks.

#### Experiment 3: Effect of Geranylacetone, Fuscumol and α-terpinene on Trap Catch

The objective of this experiment was to test for effects of the minor component, α-terpinene (AT), on trap catch of *A. ferus*. We did not test the effect of *p*-mentha-1,3,8-triene on trap catches because this compound is very unstable when exposed to oxygen (Birch and Rao 1969). There were eight pheromone treatments: (1) α-terpinene; (2) (*E*)*-*geranylacetone; (3) racemic (*E*)*-*fuscumol; (4) racemic (*E*)*-*fuscumol + α-terpinene; (5) (*E*)*-*geranylacetone + *α*-terpinene; (6) (*E*)*-*geranylacetone + racemic (*E*)*-*fuscumol; (7) (*E*)*-*geranylacetone + racemic (*E*)*-*fuscumol + α-terpinene; and (8) unbaited control. The experiment was conducted in a *P. radiata* plantation forest located in Chaney’s Forest (latitude 43°25’18.26"S longitude 172°39’57.24"E, Rayonier Matariki Forests, Christchurch, New Zealand) in a 5-month-old cutover forest site from February 2 to March 30, 2023. Treatments were replicated eight times in randomized complete blocks and deployed in an 8 × 8 formation grid (64 total traps). All lures were replaced every 4 weeks.

### Data Analyses

#### Headspace Gas Volatile Collection

**Fig. 1:**
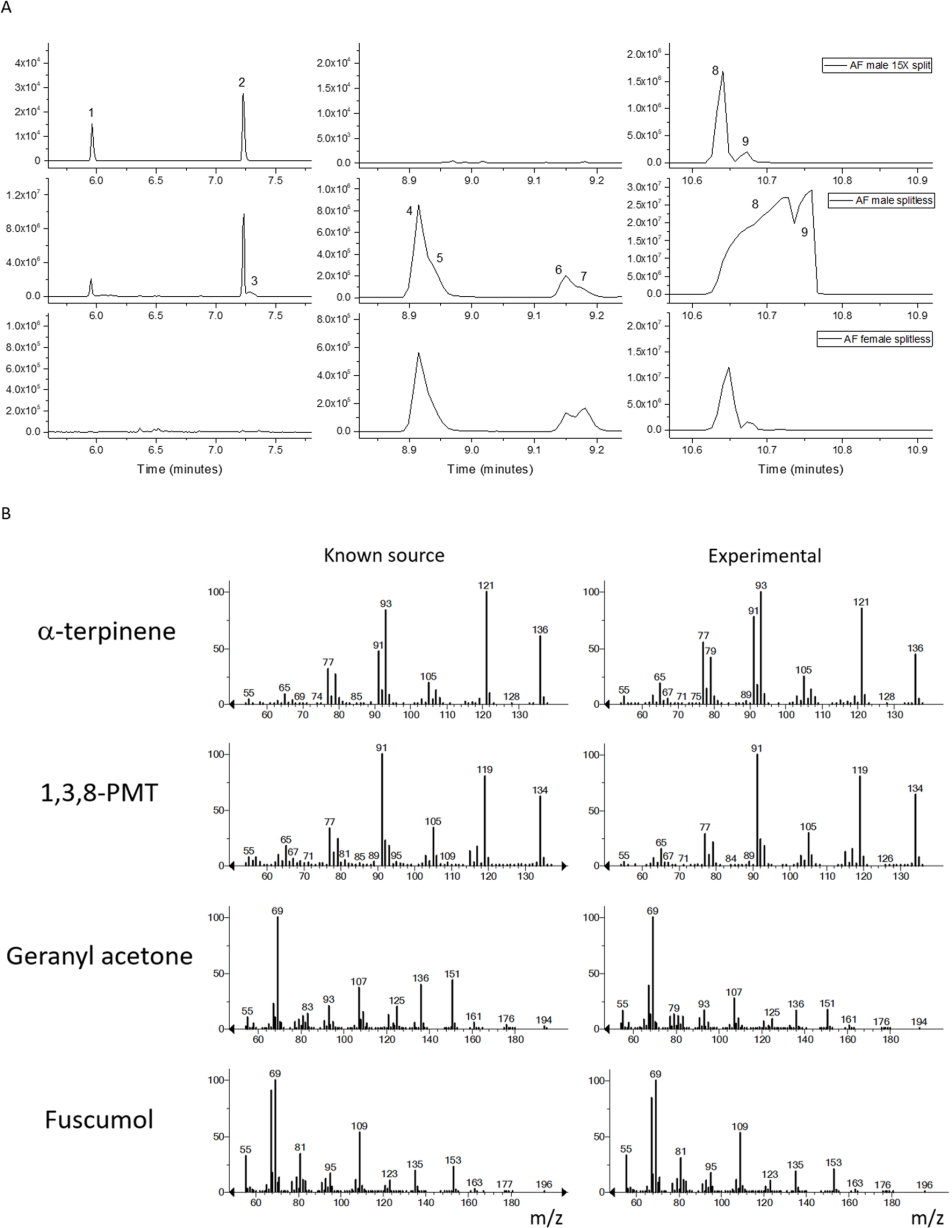
Gas chromatography-mass spectrometry (GC-MS) analyses of 24-h collections of volatiles from *Arhopalus ferus*. A) GC traces of males 15X split mode (top), males splitless mode (middle), and females splitless mode (bottom). Note difference in scale of total ion current on the y axes. B) Mass spectra. Major components were geranylacetone (8) and fuscumol (9) observed in males and in much smaller amounts in females; minor components were α-terpinene (1) and *p*-mentha-1,3,8-triene (2) observed in males only. Traces of 3-ethyl acetophenone (4), 4-ethyl acetophenone (6), unknown (3), unknown (5), and siloxane (7) were observed in male samples as well as some control samples

Gas volatile collections were analysed on an Agilent 6890 gas chromatograph (Agilent Technologies, Santa Clara CA) coupled to a 5973 N mass selective detector (MSD) fitted with an Agilent J&W DB5-MS UI column (25 m, 0.25 mm ID, 0.25 μm film, part number 122-5532UI). The inlet temperature was maintained at 250 °C in split or splitless mode. When in split mode, a 15:1 ratio was set. Helium was used as the carrier gas and set to constant flow at 1.0mL/min. The oven initial temperature was 70 °C, held for 3 min, then increased to 245 °C at a rate of 15°/min and held for 25 min, then increased to 310 °C at a rate of 25°/min and held for a final 5 min for a total run time of 47.2 min. The transfer line was set to 280 °C and the electron impact source and quadrupole temperatures were set to 230 °C and 150 °C respectively. A solvent delay of 6 min was applied to the MSD. Mass spectra and retention times of the identified compounds were confirmed with injections of authentic standards (Fig. 1B).

#### *Arhopalus ferus* Antennal Responses to a Range of Doses of Pheromone Compounds

EAG peaks were assessed using EAGPro (Syntech, NL). Dose-specific EAG responses were normalised by dividing them by the equivalent control (hexane) response. To account for the repeated measures on the same individual, data were analyzed using a linear mixed model with log transformed normalised antennal response (mV) as the response variable, dose, compound, sex, and the interaction between dose and compound as fixed factors, and ‘block’ (the series of puffs of one compound in different dosages) nested within ‘individual’ (block/individual) as a random factor. Data analysis was performed in R version 4.2.2 (R Development Core Team 2022) with the packages ‘lme4’ (Bates et al. 2014), ‘nlme’ (Pinheiro and Bates 2000; Pinheiro et al. 2022) and ‘lmerTest’(Kuznetsova et al. 2017). Type III ANOVA with Satterthwaite’s method derived from the mixed linear model was used to determine significance of the fixed factors.

Because the proportion of a given dose that reaches the antenna during a stimulus puff will vary among compounds according to their vapour pressures, post-hoc contrasts that compare mean antennal responses between compounds may be misleading, even when applied at the same “dose”. Thus, we first compared mean antennal response only among doses within each compound, and not between compounds, using sequential Sidak post-hoc contrasts in the packages ‘emmeans’ (Lenth 2023) and ‘multcomp’ (Hothorn et al. 2008). Then, exclusively for data on antennal response to racemic (*E/Z*)-fuscumol, (*R*)*-*(*E*)-fuscumol, and (*S*)*-*(*E*)-fuscumol (which have identical vapour pressures) we ran a similar model with dose, compound, and dose-compound interaction as fixed factors and block/individual as the random factor (Table 2); sex was not included because it had no significant effects on antennal response in the first analysis (Table 1). Then sequential Sidak post-hoc contrasts were used to compare mean response to the different fuscumol treatments when applied at the same dose.

**Table 1:** The effects of compound, dose, sex, and interactions between compound and dose on the log transformed, normalized electroantennogram (EAG) responses (log(mV)) of antennae excised from adults of the burnt pine longhorn beetle, *Arhopalus ferus.* Results (*P*) of the Type III ANOVA with Satterthwaite’s method derived from the mixed linear model

Factor	df	F	*P*
Compound	6, 204	11.22	< **0.0001**
Dose	3, 56	11.26	< **0.0001**
Sex	1, 15	1.14	0.300
Compound x Dose	18, 211	1.85	**0.021**

#### Field Trapping Experiments

For each experiment, the total numbers of *A. ferus* (along with distinct female and male counts) captured per trap over the entire 6–8 week trapping period were analysed independently using generalised linear models with the PROC GLIMMIX package (SAS Institute Inc 2002–2012). A very few traps (10 of 960, about 1%) were found to be damaged or on the ground during a weekly trap check. Missing values for these 10 completely random occurrences (confirmed by Little (1988) test statistic available in the ‘naniar’ package (Tierney and Cook 2018)), were imputed for the given weeks and treatments using the expectation-maximization algorithm (‘missMethods’ package (Rockel 2022)), appropriate when missingness is minimal (Little et al. 2014). This retained the balanced and complete nature of the experimental design and minimized the risk of distorting inferences (Łopucki et al. 2022). The same method was applied to impute the corresponding male/female catch counts of those traps. Imputed values were rounded to the nearest integer to account for the count nature of the data.

All models, one for *A. ferus* counts, and one for each of female and male counts, were based on the factorial design used in each experiment. Experiment 1 was based on a 2 × 2 × 2 factorial design, with each factor representing one of the three pheromone components (FA, Ra, GA), each with two levels (denoting presence or absence) and interaction terms. Experiment 2 was based on a 4 × 2 factorial design, using factor F with the four fuscumol levels (none, R, Ra, and S), and factor GA with two levels (presence or absence of GA) and a term for their interaction (F*GA). Experiment 3 was based on a 2 × 2 × 2 factorial design similar to Experiment 1, with each factor representing one of the three pheromone components, (Ra, GA, and AT), each with two levels (denoting presence or absence) and interaction terms. Block was included as a random effect in each model to reflect the study design.

All models were run with Gaussian, Poisson, and negative binomial distributions and compared using the corrected Akaike Information Criterion value (Akaike 1974; Hurvich and Tsai 1993) to select the model with best fit (i.e. lowest AICc value). ANOVA was used to determine the significance of factors in the best-fitting model. Finally, the least square means of catch in traps baited with each individual lure combination were compared with those in unbaited control traps using Dunnett’s test. For each field trapping experiment, the effect of explanatory variables on the sex ratio of *A. ferus* (i.e., number of males / number of total *A. ferus* captured per trap over the entire experiment) was analysed using generalized linear mixed models with a binomial distribution (SAS PROC GLIMMIX).

## Results

### Headspace Gas Volatile Collections

All GC profiles of headspace volatiles collected from *A. ferus* male effluvia showed two dominating peaks, with the first larger peak identified as geranylacetone and the second smaller peak identified as fuscumol; the ratio of geranylacetone to fuscumol was about 12:1 (Figs. 1A, B, Supplementary Fig. S2). The same peaks were also present in headspace volatiles collected from female effluvia but in much smaller amounts, i.e., average male volatile samples contained 36 times and 115 times the amount of geranylacetone and fuscumol, respectively, present in female volatile samples (Fig. 1, Supplementary Figs. S2–S4). The GC profiles from *A. ferus* males also contained two additional smaller peaks not observed in female volatiles, identified as *p*-mentha-1,3-diene (i.e., α-terpinene) and *p*-mentha-1,3,8-triene (PMT). The mass spectra of all four compounds matched those of authentic standards (Fig. 1B). We also detected traces of 3- and 4-ethyl acetophenone in samples from both sexes; however, these were also observed in one of the control samples so were suspected as contaminants (Supplementary Fig. S2). Fuscumol acetate was not identified in any of the effluvia samples from either sex.

### *Arhopalus ferus* Antennal Responses to a Range of Doses of Pheromone Compounds

**Fig. 2:**
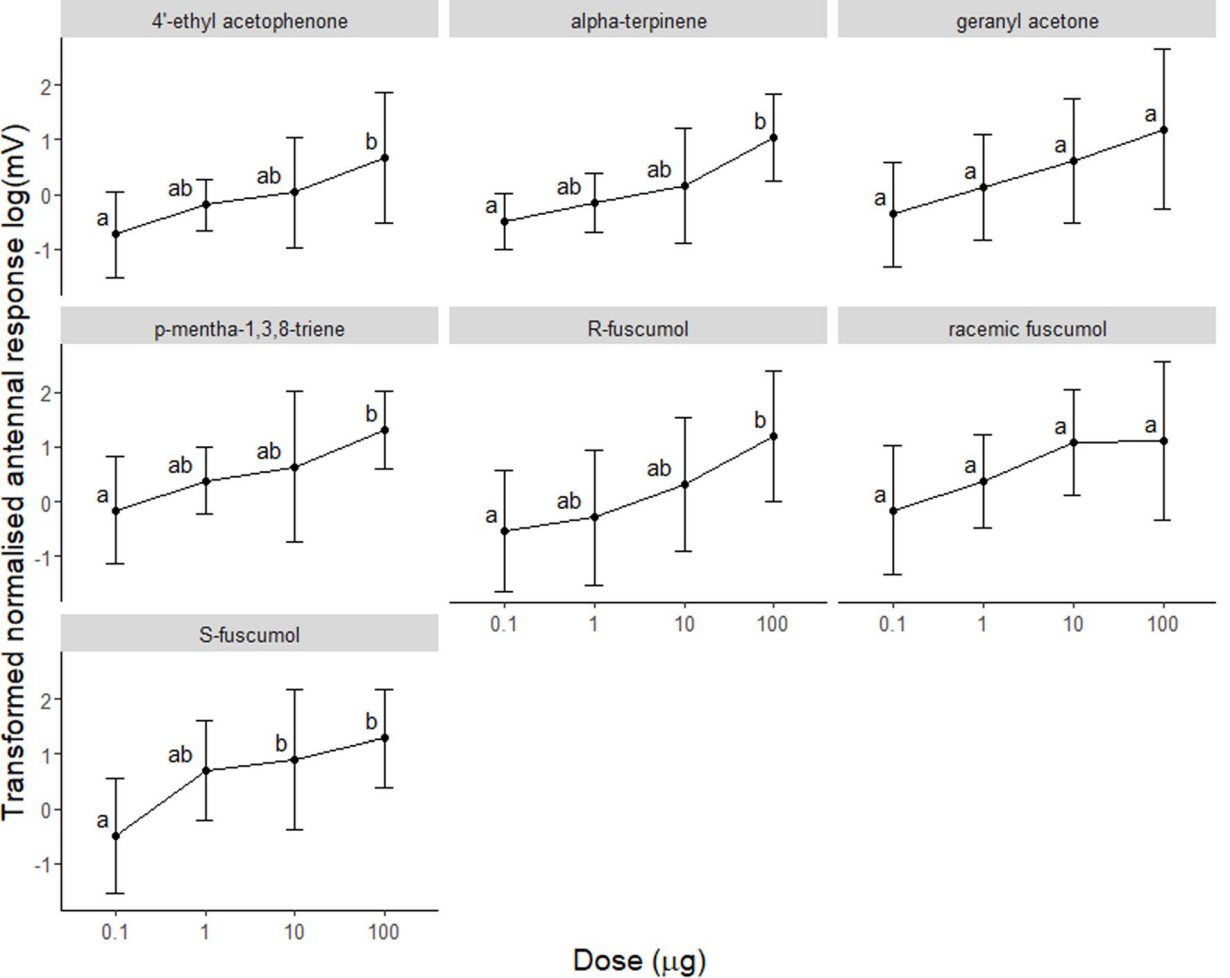
Mean values of transformed normalised antennal response (log(mv)) of *A. ferus* (sexes pooled) to increasing doses of compounds applied to filter paper and tested by EAG puffs. Within each test compound, different letters denote significantly different antennal response among doses (Sequential Sidak post-hoc contrasts tests, α = 0.05)

Normalised laboratory EAG response peaks by *A. ferus* to individual 0.3 s pheromone puffs were visually detectable above background noise at doses as low as 0.1 µg (Fig. 2). At lower than 0.1 µg dose, the antennal response was indistinguishable from the responses to hexane, our control (i.e., values of normalised EAG response ≈ 1). The generalized linear model showed that antennal responses were significantly affected by dose, compound, and dose-compound interaction, but not by *A. ferus* sex (Table 1). There was a significant positive response to increasing dose for all compounds tested except racemic fuscumol and geranylacetone (Fig. 2).

Results were similar in the second model that analysed responses only to the fuscumol compounds, with significant effects of dose, compound, and dose-compound interaction (Table 2). Antennal responses did not differ among the fuscumol compounds at the 0.1 ug or 100 ug doses. However, at 1 ug doses, mean antennal responses were significantly greater to *S*-(*E*)-fuscumol (*P* = 0.0003) and racemic (*E/Z*)-fuscumol (*P* = 0.016) than to *R*-(*E*)-fuscumol (Supplementary Table S2). Results were similar at the 10 ug doses, with significantly greater responses to racemic (*E/Z*)-fuscumol (*P* = 0.003) and marginally significantly greater responses to (*S*)-(*E*)-fuscumol (*P* = 0.06) than to (*R*)-(*E*)-fuscumol (Supplementary Table S2).

**Table 2:** The effects of selected compounds (racemic (*E/Z*)-fuscumol, (*R*)*-*(*E*)-fuscumol, (*S*)*-*(*E*)-fuscumol), dose and interactions between compound and dose on the log transformed, normalized electroantennogram (EAG) responses (log(mV)) of antennae excised from adults of the burnt pine longhorn beetle, *Arhopalus ferus.* Results (*P*) of the Type III ANOVA with Satterthwaite’s method derived from the mixed linear model

Factor	df	F	*P*
Compound	2,88	7.82	< **0.0007**
Dose	3, 54	9.22	< **0.0001**
Compound x Dose	6, 91	2.55	**0.025**

### Field Trapping Experiments

We trapped totals of 351 (244 ♂; 107♀), 966 (666♂; 300♀), and 593 (419♂; 174♀) *A. ferus* adults in Experiments 1, 2 and 3, respectively. The overall sex ratio was significantly male-biased (χ^2^ ≥ 53; *P* < 0.0001) in each experiment with about 70% males and 30% females.

#### Experiment 1: Effect of Racemic Fuscumol (Ra), Fuscumol Acetate (FA), and Geranylacetone (GA) on Trap Catch

**Fig. 3:**
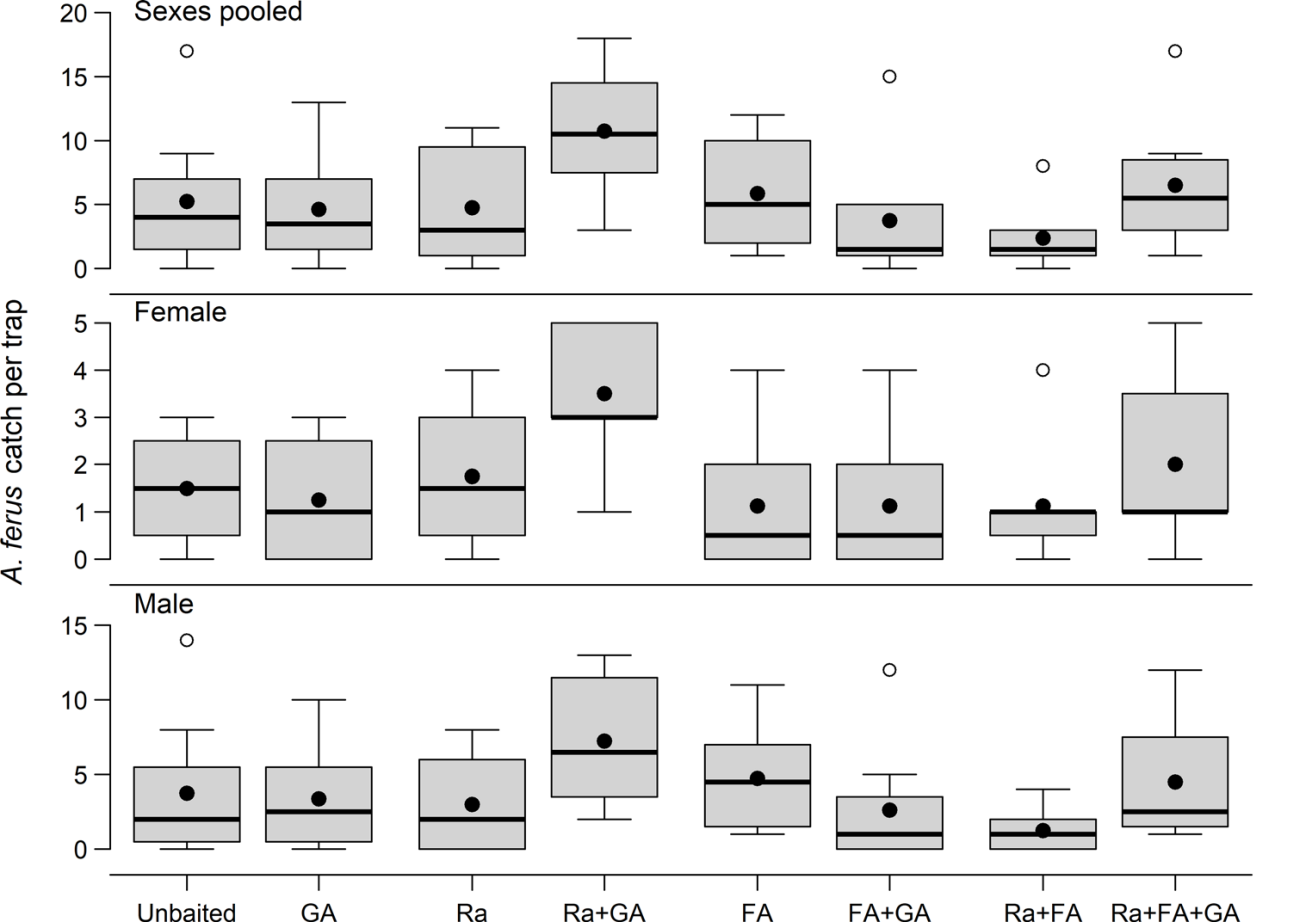
Frequency distributions (box plots) of *Arhopalus ferus* [pooled sexes, female, male] captured in flight intercept traps in a 2 × 2 × 2 factorial experiment designed to test the effects of baiting traps with all combinations of racemic (*E/Z*)-fuscumol (Ra), racemic (*E/Z*)-fuscumol acetate (FA), and (*E/Z*)-geranylacetone (GA), each with two levels (present vs. absent), in a *Pinus radiata* plantation (43°25’19” S; 172°40’43” E), Rayonier Matariki Forests, Christchurch, New Zealand, from 9 February to 16 March 2021.Treatment means are indicated by black dots. Outliers are indicated as circles. Note the difference in scale on the y axes. Treatments shaded green differed significantly from unbaited controls (Dunnett’s test, α = 0.05)

When presented alone, none of the compounds significantly affected mean catch of male- or total *A. ferus* (*F* ≤ 2.21, *P* ≥ 0.14) but Ra positively affected mean catch of females (*F* = 4.51, *P* = 0.04) and reduced male bias in the sex ratio (*F* = 4.13, *P* = 0.047) (Table 3). Results suggested that attraction of *A. ferus* required the presence of both Ra and Ga and that FA had no effect (Table 3; Fig. 3). Furthermore, the interaction between Ra and GA was significant for mean catch of males (*F* = 7.67, *P* = 0.008) and total *A. ferus* (*F* = 7.60, *P* = 0.008) but not females (*F* = 3.07, *P* = 0.09) (Table 3). However, none of the seven individual lure combinations had mean trap catches significantly different from that in unbaited traps (Dunnett’s test, *P >* 0.05).

**Table 3:** Results of a 2 × 2 × 2 factorial experiment testing effects of racemic (*E/Z*)-fuscumol (Ra), racemic (*E/Z*)-fuscumol acetate (FA), and (*E/Z*)-geranylacetone (GA), each with two levels (present vs. absent), on mean catch per trap of male, female and total *A. ferus*, and sex ratio in flight intercept traps in a *Pinus radiata* plantation at Chaney’s forest (43°25’19” S; 172°40’43” E), Rayonier Matariki Forests, Christchurch, New Zealand, 9 February to 16 March 2021. Values in bold font indicate statistically significant effects (α = 0.05). Results (*P*) of the Type III ANOVA from the linear mixed model (SAS PROC GLIMMIX)

Factor		Males		Females		Sexes pooled		Sex ratio	
	df*	F	*P*	F	*P*	F	*P*	F	*P*
Ra	1, 49	0.01	0.92	4.51	**0.039**	0.34	0.56	6.04	**0.02**
FA	1, 49	1.75	0.17	2.83	0.10	2.46	0.12	0.02	0.88
GA	1, 49	2.01	0.16	1.72	0.20	2.00	0.16	0.27	0.60
Ra*FA	1, 49	1.97	0.17	0.54	0.47	2.28	0.21	0.01	0.92
Ra*GA	1, 49	9.08	**0.004**	3.07	0.09	8.51	**0.005**	1.38	0.25
FA*GA	1, 49	0.24	0.62	0.01	0.94	0.10	0.75	0.48	0.49
Ra*FA*GA	1, 49	1.19	0.28	0.13	0.72	0.47	0.50	1.13	0.29

* degrees of freedom for sex ratio model were 1, 44

#### Experiment 2: Effect of Fuscumol Chirality and Geranylacetone on Trap Catches

**Fig. 4:**
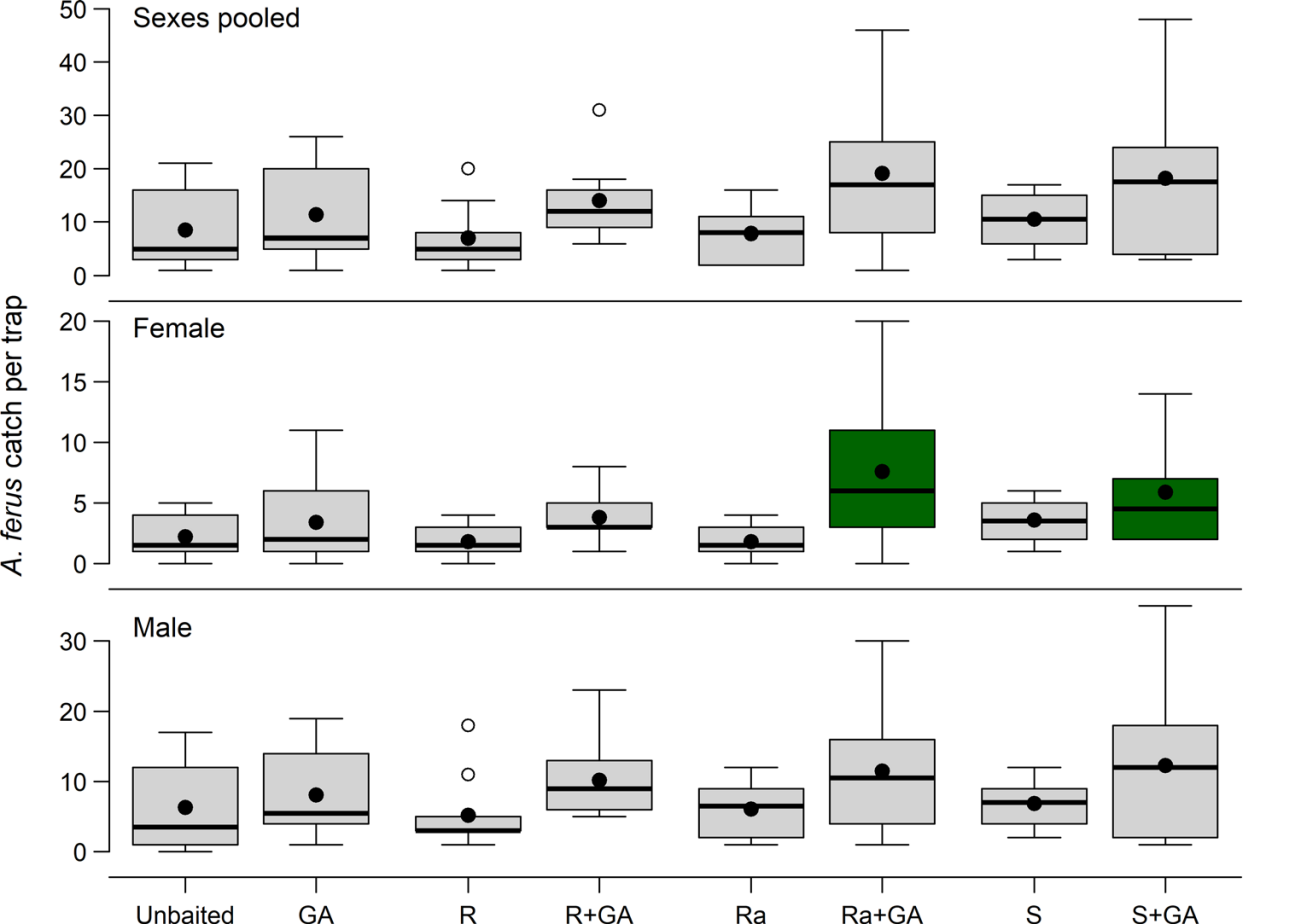
Frequency distributions (box plots) of *Arhopalus ferus* [pooled sexes, female, male] captured in flight intercept traps in a 4 × 2 factorial experiment designed to test the effects of baiting traps with all combinations of fuscumol (F) [4 levels: no fuscumol, (*R*)-(*E*)-fuscumol (R), racemic (*E*)-fuscumol (Ra), or (*S*)-(*E*)-fuscumol (S)] and geranylacetone (GA) (absent vs. present), in a *Pinus radiata* plantation forest located in Bottle Lake Forest Park (43°26’43”S, 172°41’57”E, Rayonier Matariki Forests, Christchurch, New Zealand) from 13 January to 7 April 2022. Treatment means are indicated by black dots. Outliers are indicated as circles. Note the difference in scale on the y axes. Treatments shaded green differed significantly from unbaited controls (Dunnett’s test, α = 0.05)

In contrast to results of Experiment 1, (*E*)-geranylacetone significantly affected mean catch of *A. ferus* males and females (*F*_1, 72_ ≥ 9.68, *P* ≤ 0.003) whereas fuscumol had only a near-significant effect on catch of females (*F*_1, 72_ = 2.60, *P* = 0.06) and no effect on catch of males (Table 4). Sex ratio was not significantly affected by fuscumol chirality or geranylacetone (Table 4). However, traps baited with the binary combination of geranylacetone plus either (*S* )-fuscumol or racemic fuscumol were the only treatments that captured significantly more female *A. ferus* than unbaited traps (Dunnett’s test, *P ≤* 0.05) (Fig. 4).

**Table 4:** Results of a 4 × 2 factorial experiment testing effects of fuscumol (F) [4 levels: (*R*)-(*E*)-fuscumol, racemic (*E*)-fuscumol, (*S*)-(*E*)-fuscumol, or no fuscumol) and geranylacetone (GA) (present vs. absent) on catches of male, female and total *A. ferus* in flight intercept traps, conducted in a *P. radiata* plantation forest located in Bottle Lake Forest park (43°26’43”S, 172°41’57”E), Rayonier Matariki forests, Christchurch, New Zealand, from 13 January to 7 April 2022. Values in bold font indicate statistically significant effects (α = 0.05). Results (*P*) of the Type III ANOVA from the linear mixed model (SAS PROC GLIMMIX)

Factor		Males		Females		Sexes pooled		Sex ratio	
	df*	F	*P*	F	*P*	F	*P*	F	*P*
F	3, 72	0.68	0.57	2.60	0.06	1.68	0.18	0.91	0.44
GA	1, 72	9.45	**0.003**	19.6	**<0.0001**	15.6	**0.0002**	3.13	0.08
F*GA	3, 72	0.44	0.73	1.77	0.16	0.82	0.49	1.94	0.13

* degrees of freedom for sex ratio model were 3, 63 for F and F*GA; 1, 63 for GA

#### Experiment 3: Effect of Geranylacetone, Fuscumol and α-terpinene on Trap Catch

**Fig. 5:**
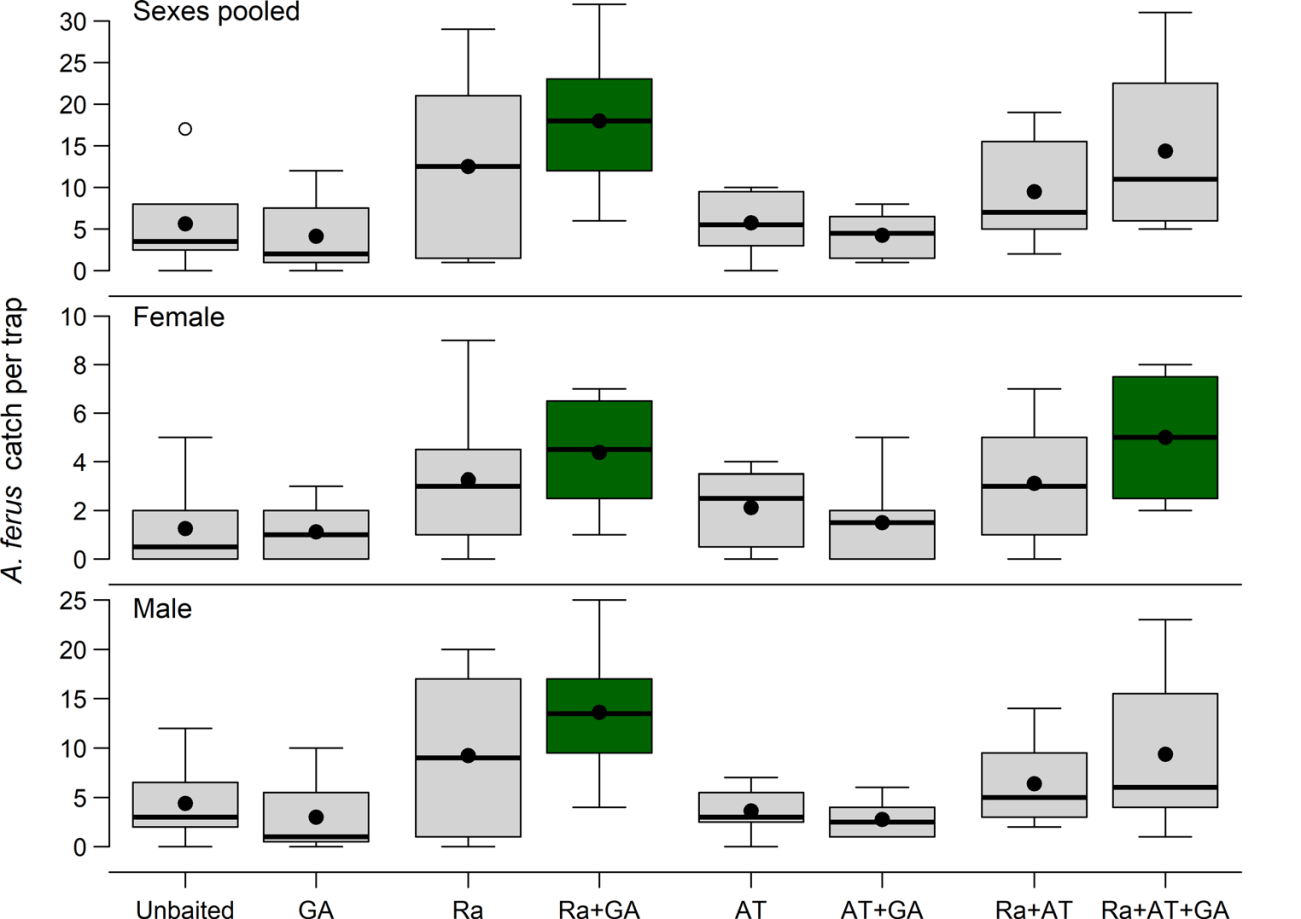
Frequency distributions (box plots) of *Arhopalus ferus* [pooled sexes, female, male] captured in flight intercept traps in a 2 × 2 × 2 factorial experiment designed to test the effects of baiting traps with all combinations of racemic (*E*)-fuscumol (Ra), (*E)-*geranylacetone (GA), and α-terpinene (AT), each with two levels (present vs. absent), in a *Pinus radiata* plantation forest (43°25’19” S, 172°40’43” E), Rayonier Matariki Forests, Christchurch, New Zealand, 2 February to 30 March 2023. Treatment means are indicated by black dots. Outliers are indicated as circles. Note the difference in scale on the y axes. Treatments shaded green differed significantly from unbaited controls (Dunnett’s test, α = 0.05)

Mean catches of *A. ferus* males and females were significantly affected by the presence or absence of racemic (*E*)-fuscumol (Ra) (*F*_1, 49_ = ≥ 25.8, *P* < 0.0001) but not (*E*)-geranylacetone (*F*_1, 49_ = ≤ 0.16, *P* ≥ 0.69), with the interaction between racemic (*E*)-fuscumol and geranylacetone (Ra*GA) being weakly significant for both sexes (*F*_1, 49_ ≥ 2.89; *P* ≤ 0.10) (Table 5). Traps baited with the binary combination of racemic fuscumol plus (*E*)-geranylacetone had significantly greater mean catches of *A. ferus* males (13.63 ± 2.27), females (4.38 ± 0.80) and both sexes combined (18.0 ± 2.94) than did unbaited traps. Traps baited with the ternary combination of racemic fuscumol plus geranylacetone plus α-terpinene also captured significantly more females than did unbaited traps, but mean catch in all other lure treatments did not differ significantly from that in unbaited traps (Dunnett’s test, *P* ≤ 0.05, Fig. 5). α-Terpinene did not affect mean catch of either sex (Table 5; Fig. 5) but significantly affected the sex ratio of *A. ferus* captured (Table 5). Traps baited with α-terpinene were less male biased (65.3 ± 0.03% males) than traps without it (72.9 ± 0.05% males). The sex ratio was least male biased in traps baited with all three compounds (59.0 ± 0.07% males) and most male-biased in unbaited traps (80.9 ± 0.07% males).

**Table 5:** Results of a 2 × 2 × 2 factorial experiment testing effects of racemic (*E*)-fuscumol (Ra), (*E*)- geranylacetone (GA), and α-terpinene (AT), each with two levels (present vs. absent), on mean catch per trap and sex ratio of *A. ferus* in flight intercept traps in a *Pinus radiata* plantation in Chaney’s forest (43°25’18.26"S, 172°39’57.24"E), Rayonier Matariki Forests, Christchurch, New Zealand, 2 February–30 March 30, 2023. Values in bold font indicate statistically significant differences (α = 0.05). Results (*P*) of the Type III ANOVA from the linear mixed model (SAS PROC GLIMMIX)

Factor		Males		Females		Sexes pooled		Sex ratio	
	df*	F	*P*	F	*P*	F	*P*	F	*P*
Ra	1, 49	25.8	**< 0.0001**	32.1	**< 0.0001**	30.8	**< 0.0001**	0.00	0.99
GA	1, 49	0.01	0.90	0.16	0.69	0.06	0.80	0.10	0.75
AT	1, 49	1.29	0.26	1.58	0.21	0.15	0.70	**5.60**	**0.02**
Ra*GA	1, 49	3.24	0.08	2.89	0.10	3.44	0.07	0.14	0.71
Ra*AT	1, 49	0.71	0.40	1.20	0.28	1.16	0.29	0.02	0.89
GA*AT	1, 49	0.03	0.87	0.00	0.96	0.01	0.94	0.01	0.93
Ra*GA*AT	1, 49	0.03	0.86	0.42	0.52	0.01	0.94	0.44	0.51

* degrees of freedom for sex ratio model were 1, 46

## Discussion

We provide evidence that (*E*)-fuscumol and geranylacetone are likely components of the aggregation-sex pheromone of the burnt pine longhorn beetle, *A. ferus*. Both compounds were identified in volatiles emitted by adult beetles, elicited a positive dose-dependent response in antennae of both sexes, and when combined on flight intercept traps, had significant positive effects on catches of *A. ferus* females in two of three field experiments. These results suggest that flight intercept traps baited with the combination of racemic (*E*)-fuscumol plus geranylacetone may be an effective survey tool for *A. ferus.* However, because significant attraction to the binary pheromone combination was evident in only one (males) or two (females) of our three trapping experiments, further research will be necessary to confirm this, as discussed below.

GC-MS analysis of effluvia collected from adult *A. ferus* indicated (*E*)-fuscumol (chirality undetermined) and geranylacetone were emitted by both sexes, but in much smaller amounts by females. Both fuscumol and geranylacetone are common pheromone structures for species in the subfamily Spondylidinae (Halloran et al. 2018; Hanks and Millar 2016; Silk et al. 2007; Sweeney et al. 2010) and were also identified in male effluvia of the congener *A. rusticus* (Žunič-Kosi et al. 2019). Presence of fuscumol and geranylacetone in female effluvia differs from previous studies of Spondylidinae species in which these pheromones were only detected in males (Halloran et al. 2018; Silk et al. 2007, 2010; Sweeney et al. 2010; Žunič-Kosi et al. 2019) and we report this with caution. The amounts of pheromones in female effluvia were a tiny fraction (about 2%) of those observed in males, and female emission of pheromones does not fit the paradigm for longhorn beetles in the Spondylidinae, Cerambycinae, and Lamiinae (Hanks and Millar 2016). It is quite possible that the small amounts of fuscumol and geranylacetone observed in female samples were an artefact or a cross-contaminant and were in fact produced by males. Depending on the accuracy of the sex determinations on the evening of collection, it is possible that some males may have inadvertently been housed with females and male-emitted pheromones may have adhered to the bodies of females prior to use in volatile collections. Yasui et al. (2007) showed that sesquiterpenes released from host plants during adult feeding were adsorbed onto the waxy cuticle of *Anoplophora malasiaca* (Thomson) adults and subsequently slowly released from the wax layer.

The minor components, α-terpinene and *p*-mentha-1,3,8-triene, were identified in male but not female effluvia. α-Terpinene elicited much lower antennal responses than geranylacetone or fuscumol and did not affect absolute numbers of *A. ferus* captured in traps. However, α-terpinene significantly affected the sex ratio of *A. ferus*, reducing the male bias in trap catches, suggesting it may be more behaviourally active in females than males. However, its precise role on *A. ferus* female attraction warrants further investigation. We did not test the effect of *p*-mentha-1,3,8-triene on trap catch, mainly due to its instability on exposure to oxygen (Birch and Rao 1969). *p*-Mentha-1,3,8-triene elicited antennal responses similar to those of geranylacetone, suggesting that its role in pheromone-mediated behaviour of *A. ferus* requires further investigation. However, *p*-mentha-1,3,8-triene is unstable when expose to oxygen (Birch and Rao 1969) so it is possible that the antennal response was elicited by a oxidation product. Addition of the antioxidant BHT (butylated hydroxytoluene) to lures might stabilize *p*-mentha-1,3,8-triene and allow for field testing, as previously done for *p*-mentha-1,3-diene-8-ol (Collignon et al. 2019.

Both α-terpinene and *p*-mentha-1,3,8-triene were identified in the male effluvia of two other species of longhorn beetles, *Paranoplium gracile* and *Eudistenia costipennis* (Cerambycinae: Oemini) (Collignon et al. 2019) and a compound with a similar 1-methyl-4-alkylcyclohexane backbone, α-terpineol, is emitted by males of *Megacyllene caryae* (Gahan) (Lacey et al. 2008) and *M.* *antennata* (White) (Mitchell et al. 2018) (Cerambycinae: Clytini). The presence of the antennally-active terpenoids, α-terpinene and *p*-mentha-1,3,8-triene, in the male effluvia of a longhorn beetle in the Spondylidinae subfamily adds further support to the hypothesis of Collignon et al. (2019) that 1-methyl-4-alkylcyclohexane may represent yet another generic pheromone motif in the Cerambycidae.

It is generally accepted that the primary function of male-produced pheromones in the Cerambycinae, Lamiinae, and Spondylidinae is to attract both sexes to the same location and thereby increase the probability of mating success, which is why they are referred to as aggregation-sex pheromones (Millar and Hanks 2017). In many species of Cerambycidae, males emerge before females and are the first to arrive on the larval host tree where mating usually takes place (Hanks and Wang 2017; Linsley 1995), and this is also true for *A. ferus* (Hosking 1978). The first males that arrive on the host tree must be able to locate it independent of pheromones, likely using a combination of olfactory (host volatiles) and visual cues (Hanks and Wang 2017). Indeed, the relatively high catches of *A. ferus* in unbaited traps in our study suggests that visual cues as well as olfactory cues play a significant role in their location of suitable hosts and mating sites, as previously suggested by Brockerhoff et al. (2006) and Kerr et al. (2017). After arrival of males on a suitable host, male emission of aggregation-sex pheromones attracts both sexes (Millar and Hanks 2017).

Several studies have found a female-biased sex ratio of longhorn beetles in traps baited with aggregation-sex pheromones or the combination of aggregation-sex pheromones and host volatiles. This was true for *A. rusticus* (Žunič-Kosi et al. 2019), other Spondylidinae such as *T. fuscum* (Sweeney et al. 2010), as well as several *Monochamus* spp. in the Lamiinae (Allison et al. 2012; Pajares et al. 2010; Ryall et al. 2015; Teale et al. 2011). In *T. fuscum*, which has an unbiased sex ratio in nature (Juutinen 1955), catches were significantly female-biased in traps baited with racemic fuscumol plus host volatiles and were either unbiased or significantly male-biased in traps baited with host volatiles alone (Sweeney et al. 2010). Female-biased attraction to male-produced aggregation pheromones is also common in bark beetles (Curculionidae: Scolytinae) suggesting they may have evolved in Coleoptera with aggregation behaviours from initial roles as sex pheromones for female attraction (Raffa et al. 1993).

However, in our study the overall sex ratio of *A. ferus* captured in traps was significantly male-biased, averaging about 70% males. This is more male biased than sex ratios reported for *A. ferus* emerging from infested logs in New Zealand (66% male) (Hosking 1978), *A. syriacus* (Reitter) reared from infested logs in Australia (60% male) (Webb and Eldridge 1997), and *A. syriacus* and *A. rusticus* reared from infested logs or captured in terpene-baited traps in Argentina (50% males for both species) (Fachinetti and Grilli 2020). The predominant male-bias in the *A. ferus* sex ratio in our trapping studies suggests that further studies may be necessary to determine the role of the minor pheromone components detected in our study in the attraction of females, or that additional pheromone components are yet to be discovered. In Experiment 3 we found that traps baited with the ternary combination of racemic fuscumol, geranylacetone and α-terpinene had the least male-bias of all treatments (59% ± 6.7 SE males) whereas unbaited traps had the greatest male bias (80.9% ± 6.8 SE males). It is possible that traps baited with the quaternary pheromone blend of major and minor pheromone components that we detected from effluvia of male *A. ferus*, i.e., fuscumol, geranylacetone, α-terpinene, and *p*-mentha-1,3,8-triene, may increase catch of females and reduce male-bias in trap catch, but this remains to be tested.

We did not determine the chirality of fuscumol emitted by *A. ferus.* However, the stronger antennal response to (*S*)- than (*R*)-fuscumol at the 1 ug dose suggests that *A. ferus* may have more antennal sensilla receptive to the (*S*) vs. the (*R*) enantiomer. Experiment 2 showed that traps baited with the combination of geranylacetone plus either (*S*)-fuscumol or racemic fuscumol captured significantly more female *A. ferus* than did unbaited traps, whereas traps baited with geranylacetone plus (*R*)-fuscumol did not. These results, combined with the knowledge that (*S*)*-*and not (*R*)-fuscumol is emitted by the congeners, *A. rusticus* (Žunič-Kosi et al. 2019) and *A. productus* (Hanks and Millar 2016) as well as other species of Spondylidinae (Halloran et al. 2018; Sweeney et al. 2010), suggest that (*S*)-fuscumol may also be the enantiomer used by *A. ferus.* However, further research is necessary to confirm this. Our finding that racemic fuscumol performed as well or better than (*S*)-fuscumol when combined with geranylacetone has practical benefits for *A. ferus* monitoring because racemic fuscumol is cheaper to synthesize than pure (*S*)-fuscumol.

Males of both *A. ferus* and *A. rusticus* (Žunič-Kosi et al. 2019) emit fuscumol and geranylacetone as aggregation-sex pheromones and both species are sympatric in Europe and North Africa where they infest weakened and dying pines and other conifers (Sama 2002). This suggests there may be risk of cross-attraction and possible reductions in fitness as a result of heterospecific mating attempts, as observed for *T. fuscum* and *T. cinnamopterum* (Anderson et al. 2022). Many cerambycid species that share the same aggregation-sex pheromones are temporally isolated either by time of year or time of day when adults are active (Hanks and Millar 2013; Hanks et al. 2014) but according to (Sama 2002), adults of both *A. rusticus* and *A. ferus* are largely nocturnal and active between June and September in Europe. However, there were key differences between species in relative amounts of each compound emitted by males as well as their effects on trap catches. Integration of the peaks of male *A. ferus* effluvia in our GC-MS (15X split) analysis indicated a geranylacetone: fuscumol ratio of about 12:1 whereas Žunič-Kosi et al. (2019) found a reverse trend (1:4) with *A. rusticus*. We found that greatest catch of *A. ferus* occurred in traps baited with both geranylacetone and fuscumol whereas trap catch of *A. rusticus* was greatest in traps baited with fuscumol alone and was significantly reduced by the addition of geranylacetone (Žunič-Kosi et al. 2019). The negative effect of geranylacetone on attraction of *A. rusticus* may function as a reproductive isolating mechanism, reducing the probability of their arrival on hosts occupied by calling *A. ferus* males. However, since *A. ferus* attraction was greatest when both compounds were present, there may still be considerable risk of their attraction to calling male *A. rusticus*, depending on how critically attraction is affected by the geranylacetone: fuscumol ratio. It is possible that *A. ferus* are less attracted to the 1:4 ratio of geranylacetone: fuscumol emitted by *A. rusticus* than the 12:1 ratio emitted by conspecifics, and/or that lack of minor components such as *p*-mentha-1,3,8-triene, reduce *A. ferus* attraction, but this remains to be tested. Finally, species-specific blends of cuticular hydrocarbons act as contact pheromones for recognition of conspecific females and elicitation of copulatory behaviour in many cerambycids (Ginzel 2010; Ginzel and Hanks 2003) and this may be another precopulatory adaptation to reduce heterospecific mating attempts between *A. ferus* and *A. rusticus.*

Our results shed more light on our understanding of the pheromone chemistry of *A. ferus* and the Spondylidinae. We have shown that geranylacetone and (*E*)-fuscumol are emitted by male *A. ferus*, and that baiting traps with both compounds had significant positive effects on trap catches of females in two of three field experiments. Traps baited with both compounds show promise for the survey and monitoring of *A. ferus* but further research is necessary to demonstrate this. This should include tests of the effect of geranylacetone: fuscumol release rate ratio on *A. ferus* attraction. It is conceivable that synthetic lures that emit geranylacetone: fuscumol at the natural 12:1 ratio may attract more *A. ferus* than the 3:1 ratio that we used in our trapping experiments (see Supplementary Table S.1). The effects of adding *p*-mentha-1,3,8-triene or both minor components to traps baited with geranylacetone plus racemic fuscumol should also be determined, as should the relative efficacy of geranylacetone: fuscumol lures vs. the current *A. ferus* survey lure of α-pinene and ethanol. Although Kerr et al. (2022) found that traps baited with α-pinene and ethanol did not catch significantly more *A. ferus* than unbaited traps, a trapping experiment that directly compares that lure combination with the binary pheromone combination is necessary to determine which is more efficacious. Finally, future work should also determine whether the combination of host volatiles and aggregation-sex pheromones enhance or synergize *A. ferus* catch, as shown for several other longhorn beetle species (Nehme et al. 2009; Pajares et al. 2010; Reddy et al. 2005; Silk et al. 2007).

## Electronic Supplementary Material

Below is the link to the electronic supplementary material.

Supplementary Material 1

Supplementary Material 2

Supplementary Material 3

Supplementary Material 4

Supplementary Material 5

Supplementary Material 6

## Data Availability

Data available on request.

## References

[CR1] Akaike H (1974) A new look at the statistical model identification. IEEE T Automat Contr 19:716–723. 10.1109/TAC.1974.1100705

[CR3] Allison JD, Borden JH, Seybold SJ (2004) A review of the chemical ecology of the Cerambycidae (Coleoptera). Chemoecology 14:123–150. 10.1007/s00049-004-0277-1

[CR2] Allison J, Johnson C, Meeker J, Strom B, Butler S (2011) Effect of aerosol surface lubricants on the abundance and richness of selected forest insects captured in multiple-funnel and panel traps. J Econ Entomol 104:1258–1264. 10.1603/EC11044 21882690 10.1603/ec11044

[CR4] Allison JD, McKenney JL, Millar JG, Mcelfresh JS, Mitchell RF, Hanks LM (2012) Response of the woodborers *Monochamus carolinensis* and *Monochamus titillator* (Coleoptera: Cerambycidae) to known cerambycid pheromones in the presence and absence of the host plant volatile α-pinene. Environ Entomol 41:1587–1596. 10.1603/EN12185 23321107 10.1603/EN12185 PMC3763951

[CR5] Anderson JL, Heard SB, Sweeney J, Pureswaran DS (2022) Mate choice errors may contribute to slow spread of an invasive Eurasian longhorn beetle in North America. NeoBiota 71:71–89. 10.3897/neobiota.71.72843

[CR6] Barrington A, Logan D (2015) Ovarian development in *Arhopalus ferus* (Coleoptera: Cerambycidae). NZPP 68:451–451. 10.30843/nzpp.2015.68.5865

[CR7] Barrington A, Logan D, Connolly P (2015) A method for rearing *Arhopalus ferus* (Mulsant)(Coleoptera: Cerambycidae) larvae on a modified artificial diet. NZPP 68:353–359. 10.30843/nzpp.2015.68.5812

[CR8] Bates D, Mächler M, Bolker B, Walker S (2014) Fitting linear mixed-effects models using lme4. 10.48550/arXiv.1406.5823. arXiv preprint arXiv:14065823

[CR9] Birch A, Rao GS (1969) The synthesis of p-mentha-1,3,8-triene. Aust J Chem 22:2037–2039. 10.1071/CH9692037

[CR10] Boone CK et al (2019) *Monochamus* species from different continents can be effectively detected with the same trapping protocol. J Pest Sci 92:3–11. 10.1007/s10340-018-0954-4

[CR11] Brockerhoff EG (2009) Wood Borer and Bark Beetle Risk Analysis. Forest Biosecurity Research Council, New Zealand, p 23

[CR12] Brockerhoff EG, Jones DC, Kimberley MO, Suckling DM, Donaldson T (2006) Nationwide survey for invasive wood-boring and bark beetles (Coleoptera) using traps baited with pheromones and kairomones. Ecol Manag 228:234–240. 10.1016/j.foreco.2006.02.046

[CR13] Collignon RM, Halloran S, Serrano JM, McElfresh JS, Millar JG (2019) An unstable monoterpene alcohol as a pheromone component of the longhorned beetle *Paranoplium gracile* (Coleoptera: Cerambycidae). J Chem Ecol 45:339–347. 10.1007/s10886-019-01063-7 30854612 10.1007/s10886-019-01063-7

[CR14] Corey E, Suggs JW (1975) Method for catalytic dehalogenations via trialkyltin hydrides. J Org Chem 40:2554–2555. 10.1021/jo00905a039

[CR15] Eyre D, Haack RA (2017) Invasive cerambycid pests and biosecurity measures. Cerambycidae of the World. CRC, pp 577–632

[CR16] Fachinetti R, Grilli MP (2020) Biological traits and field distribution of introduced *Arhopalus* species in Central Argentina. Int J Pest Manag 68:274–284. 10.1080/09670874.2020.1818871

[CR17] Ginzel MD (2010) Hydrocarbons as contact pheromones of longhorned beetles (Coleoptera: Cerambycidae). Insect HC: Biol Biochem Chem Ecol :375–389

[CR18] Ginzel MD, Hanks LM (2003) Contact pheromones as mate recognition cues of four species of longhorned beetles (Coleoptera: Cerambycidae). J Insect Behav 16:181–187. 10.1023/A:1023911701159

[CR19] Grobbelaar E (2017) Longhorn beetles: the good, the bad and the ugly (Coleoptera: Cerambycidae). Paper presented at the Combined Congress of the Entomological and Zoological Societies of Southh Africa, CSIR International Convention Centre, Pretoria, South Africa

[CR20] Haack RA (2017) Cerambycid pests in forests and urban areas. In: Wang Q (ed) Cerambycidae of the world: biology and pest management. CRC, Boca Raton, FL, pp 351–407

[CR21] Halloran ST, Collignon RM, McElfresh JS, Millar JG (2018) Fuscumol and geranylacetone as pheromone components of Californian longhorn beetles (Coleoptera: Cerambycidae) in the subfamily Spondylidinae. Environ Entomol 47:1300–1305. 10.1093/ee/nvy101 29986003 10.1093/ee/nvy101

[CR22] Hanks LM, Millar JG (2013) Field bioassays of cerambycid pheromones reveal widespread parsimony of pheromone structures, enhancement by host plant volatiles, and antagonism by components from heterospecifics. Chemoecology 23:21–44. 10.1007/s00049-012-0116-8

[CR23] Hanks LM, Millar JG (2016) Sex and aggregation-sex pheromones of cerambycid beetles: Basic science and practical applications. J Chem Ecol 42:631–654. 10.1007/s10886-016-0733-8 27501814 10.1007/s10886-016-0733-8

[CR25] Hanks LM, Wang Q (2017) Reproductive biology of cerambycids. Cerambycidae of the world: biology and pest management. CRC, Boca Raton, p 28

[CR24] Hanks LM et al (2014) Seasonal phenology of the cerambycid beetles of east central Illinois. Ann Entomol Soc 107:211–226. 10.1603/AN13067 10.1603/AN13067 PMC3969037 24683267

[CR26] Hosking G (1978) *Arhopalus ferus* (Mulsant), (Coleoptera: Cerambycidae): burnt pine longhorn. Forest Research Institute, New Zealand Forest Service, New Zealand

[CR27] Hothorn T, Bretz F, Westfall P (2008) Simultaneous inference in general parametric models. Biom J 50:346–363. 10.1002/bimj.200810425 18481363 10.1002/bimj.200810425

[CR28] Hughes GP, Meier LR, Zou Y, Millar JG, Hanks LM, Ginzel MD (2016) Stereochemistry of fuscumol and fuscumol acetate influences attraction of longhorned beetles (Coleoptera: Cerambycidae) of the subfamily Lamiinae. Environ Entomol 45:1271–1275. 10.1093/ee/nvw101 27523086 10.1093/ee/nvw101

[CR29] Hurvich CM, Tsai CL (1993) A corrected akaike information criterion for vector autoregressive model selection. J Time Ser Anal 14:271–279. 10.1111/j.1467-9892.1993.tb00144.x

[CR30] Jurc M, Bojovic S, Fernández MF, Jurc D (2012) The attraction of cerambycids and other xylophagous beetles, potential vectors of *Bursaphelenchus xylophilus*, to semio-chemicals in Slovenia. Phytoparasitica 40:337–349. 10.1007/s12600-012-0234-4

[CR31] Juutinen P (1955) Zur Biologie und forstlichen Bedeutung Der Fichtenböcke (*Tetropium* Kirby) in Finnland. Suomen hyönteistieteellinen Seura 11

[CR32] Kaissling K-E (1995) 4.19. Single unit and Electrontennogram recordings in insect olfactory organs. In: Experimental cell biology of taste and olfaction: current techniques and protocols, vol 361

[CR34] Kerr JL, Kelly D, Bader MKF, Brockerhoff EG (2017) Olfactory cues, visual cues, and semiochemical diversity interact during host location by invasive forest beetles. J Chem Ecol 43:17–25. 10.1007/s10886-016-0792-x 27832345 10.1007/s10886-016-0792-x

[CR33] Kerr JL, Dickson G, O’Connor BC, Somchit C, Sweeney J, Pawson SM (2022) Effect of host volatile release rate and racemic fuscumol on trap catch of *Hylurgus ligniperda, Hylastes ater* (Coleoptera: Curculionidae), and *Arhopalus ferus* (Coleoptera: Cerambycidae). J Econ Entomol 115:168–177. 10.1093/jee/toab203 34761254 10.1093/jee/toab203

[CR35] Kuznetsova A, Brockhoff PB, Christensen RHB (2017) lmerTest package: tests in linear mixed effects models. J STAT SOFTW 82

[CR36] Lacey ES, Moreira JA, Millar JG, Hanks LM (2008) A male-produced aggregation pheromone blend consisting of alkanediols, terpenoids, and an aromatic alcohol from the cerambycid beetle *Megacyllene caryae*. J Chem Ecol 34:408–417. 10.1007/s10886-008-9425-3 18253798 10.1007/s10886-008-9425-3

[CR37] Lenth R (2023) ‘emmeans’: Estimated marginal means, aka least-squares means, R package version 1.8.5 edn

[CR38] Linit M (1988) Nemtaode-vector relationships in the pine wilt disease system. J Nematol 20:227 19290206 PMC2618795

[CR39] Linsley E (1995) The banded alder beetle in natural and urban environments (Coleoptera: Cerambycidae). Pan-Pac Entomol 71:133–134

[CR40] Little RJ (1988) A test of missing completely at random for multivariate data with missing values. J Am Stat Assoc 83:1198–1202

[CR41] Little TD, Jorgensen TD, Lang KM, Moore EWG (2014) On the joys of missing data. J Pediatr Psychol 39:151–162 23836191 10.1093/jpepsy/jst048

[CR42] Łopucki R, Kiersztyn A, Pitucha G, Kitowski I (2022) Handling missing data in ecological studies: ignoring gaps in the dataset can distort the inference. Ecol Model 468:109964

[CR43] Lynikienė J, Tamutis V, Gedminas A, Marčiulynas A, Menkis A (2021) First report of the larch longhorn (*Tetropium gabrieli* Weise, Coleoptera: Cerambycidae: Spondylidinae) on *Larix* spp. Lith Insects 12. 10.3390/insects12100911 10.3390/insects12100911 PMC8538462 34680679

[CR45] Meier LR, Zou Y, Millar JG, Mongold-Diers JA, Hanks LM (2016) Synergism between enantiomers creates species-specific pheromone blends and minimizes cross-attraction for two species of cerambycid beetles. J Chem Ecol 42:1181–1192. 10.1007/s10886-016-0782-z 27771798 10.1007/s10886-016-0782-z

[CR44] Meier LR, Millar JG, Mongold-Diers JA, Hanks LM (2019) (*S*)-Sulcatol is a pheromone component for two species of cerambycid beetles in the subfamily Lamiinae. J Chem Ecol 45:447–454. 10.1007/s10886-019-01071-7 30989491 10.1007/s10886-019-01071-7

[CR46] Meier LR, Zou Y, Mongold-Diers JA, Millar JG, Hanks LM (2020) Pheromone composition and chemical ecology of six species of cerambycid beetles in the subfamily Lamiinae. J Chem Ecol 46:30–39. 10.1007/s10886-019-01128-7 31808075 10.1007/s10886-019-01128-7

[CR48] Millar JG, Hanks LM (2017) Chemical ecology of cerambycids. In: Wang Q (ed) Cerambycidae of the World: Biology and Pest Management. CRC, Boca Raton, pp 161–208. 10.1201/b21851

[CR47] Millar J et al (2017) Identifying possible pheromones of cerambycid beetles by field testing known pheromone components in four widely separated regions of the United States. J Econ Entomol 111:252–259. 10.1093/jee/tox312 10.1093/jee/tox312 29228303

[CR49] Mitchell RF et al (2011) Fuscumol and fuscumol acetate are general attractants for many species of cerambycid beetles in the subfamily Lamiinae. Entomol Exp Appl 141:71–77. 10.1111/j.1570-7458.2011.01167.x

[CR50] Mitchell RF, Ray AM, Hanks LM, Millar JG (2018) The common natural products (S)-α-terpineol and (E)-2-hexenol are important pheromone components of *Megacyllene antennata* (Coleoptera: Cerambycidae). Environ Entomol 47:1547–1552. 10.1093/ee/nvy126 30137276 10.1093/ee/nvy126 PMC6692852

[CR51] Nehme M, Keena M, Zhang A, Baker T, Hoover K (2009) Attraction of *Anoplophora glabripennis* to male-produced pheromone and plant volatiles. Environ Entomol 38:1745–1755. 10.1603/022.038.0628 20021771 10.1603/022.038.0628

[CR52] Pajares JA, Álvarez G, Ibeas F, Gallego D, Hall DR, Farman DI (2010) Identification and field activity of a male-produced aggregation pheromone in the pine sawyer beetle, *Monochamus galloprovincialis*. J Chem Ecol 36:570–583. 10.1007/s10886-010-9791-5 20437083 10.1007/s10886-010-9791-5

[CR53] Pawson SM, Kerr JL, Kimberley M, Meurisse N, Somchit C, Wardhaugh C (2020a) Large-scale, multi-year, phenology modelling of forest insects in *Pinus radiata* plantations. J Pest Sci. 10.1007/s10340-021-01328-9

[CR54] Pawson SM, Kerr JL, Somchit C, Wardhaugh CW (2020b) Flight activity of wood-and bark-boring insects at New Zealand ports. N Z J Forest Sci 50

[CR55] Pinheiro J, Bates D (2000) Mixed-effects models in S and S-PLUS. Stat. Comput. Springer New York. 10.1007/b98882

[CR56] Pinheiro J, Bates D, R Core Team (2022) ‘nlme’: Linear and Nonlinear Mixed Effects Models, R package version 3.1–160, edn

[CR57] R Development Core Team (2022) A language and environment for statistical computing R Foundation for Statistical Computing, Vienna, Austria

[CR58] Raffa K, Phillips T, Salom S (1993) Strategies and mechanisms of host colonization by bark beetles. In: Schowalter TD, Filip GM (eds) Beetle-pathogen interactions in conifer forests. Academic, New York, pp 103–120

[CR59] Reddy GV, Fettköther R, Noldt U, Dettner K (2005) Enhancement of attraction and trap catches of the old-house borer, *Hylotrupes bajulus* (Coleoptera: Cerambycidae), by combination of male sex pheromone and monoterpenes. Pest Manag Sci 61:699–704. 10.1002/ps.1044 15776406 10.1002/ps.1044

[CR60] Rockel T (2022) missMethods: Methods for missing data. R package version 03 0 URL https://CRAN R-project org/package = missMethods

[CR61] Ryall K et al (2015) Further evidence that monochamol is attractive to *Monochamus* (Coleoptera: Cerambycidae) species, with attraction synergised by host plant volatiles and bark beetle (Coleoptera: Curculionidae) pheromones. Can Entomol 147:564–579. 10.4039/tce.2014.67

[CR62] Sama G (2002) Atlas of the Cerambycidae of Europe and the Mediterranean Area, vol 1. Kabourek

[CR63] SAS Institute Inc (2002–2012) SAS/STAT software, 9.4 of the SAS System for Windows edn., Cary, NC, USA

[CR64] Schroeder M, Cocoş D, Johansson H, Sweeney J (2021) Attraction of the cerambycid beetles *Tetropium gabrieli, T. Castaneum* and *T. fuscum* to pheromones and host tree volatiles. Agric Entomol 23:203–211. 10.1111/afe.12422

[CR66] Silk PJ, Sweeney J, Wu J, Price J, Gutowski JM, Kettela EG (2007) Evidence for a male-produced pheromone in *Tetropium fuscum* (F.) and *Tetropium cinnamopterum* (Kirby) (Coleoptera: Cerambycidae). Naturwissenschaften 94:697–701. 10.1007/s00114-007-0244-0 17429600 10.1007/s00114-007-0244-0

[CR65] Silk PJ, Lemay MA, LeClair G, Sweeney J, MaGee D (2010) Behavioral and electrophysiological responses of *Tetropium fuscum* (Coleoptera: Cerambycidae) to pheromone and spruce volatiles. Environ Entomol 39:1997–2005. 10.1603/EN10156 22182567 10.1603/EN10156

[CR67] Sweeney JD, Silk PJ, Gutowski JM, Wu J, Lemay MA, Mayo PD, Magee DI (2010) Effect of chirality, release rate, and host volatiles on response of *Tetropium fuscum* (F.), *Tetropium cinnamopterum* (Kirby), and *Tetropium castaneum* (L.) to the aggregation pheromone, fuscumol. J Chem Ecol 36:1309–1321. 10.1007/s10886-010-9876-1 21046204 10.1007/s10886-010-9876-1

[CR68] Teale SA et al (2011) A male-produced aggregation pheromone of *Monochamus alternatus* (Coleoptera: Cerambycidae), a major vector of pine wood nematode. J Econ Entomol 104:1592–1598. 10.1603/EC11076 22066189 10.1603/ec11076

[CR69] Tierney NJ, Cook DH (2018) Expanding tidy data principles to facilitate missing data exploration, visualization and assessment of imputations. arXiv Preprint arXiv :180902264

[CR70] Wang Q, Leschen RA (2003) Identification and distribution of *Arhopalus* species (Coleoptera: Cerambycidae: Aseminae) in Australia and New Zealand. New Z Entomol 26:53–59

[CR71] Webb G, Eldridge R (1997) *Arhopalus syriacus* (Reitter) (Coleoptera: Cerambycidae): a potential economic pest of *Pinus* in Australia, with notes on its biology and distribution. Aust for 60:125–129. 10.1080/00049158.1997.10674707

[CR72] Yasui H et al (2007) Host plant chemicals serve intraspecific communication in the white-spotted longicorn beetle, *Anoplophora malasiaca* (Thomson)(Coleoptera: Cerambycidae). Appl Entomol Zool 42:255–268. 10.1303/aez.2007.255

[CR73] Žunič-Kosi A, Stritih-Peljhan N, Zou Y, McElfresh JS, Millar JG (2019) A male-produced aggregation-sex pheromone of the beetle *Arhopalus rusticus* (Coleoptera: Cerambycidae, Spondylinae) may be useful in managing this invasive species. Sci Rep 9:1–10. 10.1038/s41598-019-56094-7 31863031 10.1038/s41598-019-56094-7 PMC6925271

