# Supplementary figures and images for: Exploring the Nature of Arhopalus ferus (Coleoptera: Cerambycidae: Spondylidinae) Pheromone Attraction
Source: J Chem Ecol. 2024 Jun 6;50(12):904–19. doi: 10.1007/s10886-024-01508-8 (PMC11717888; doi:10.1007/s10886-024-01508-8)

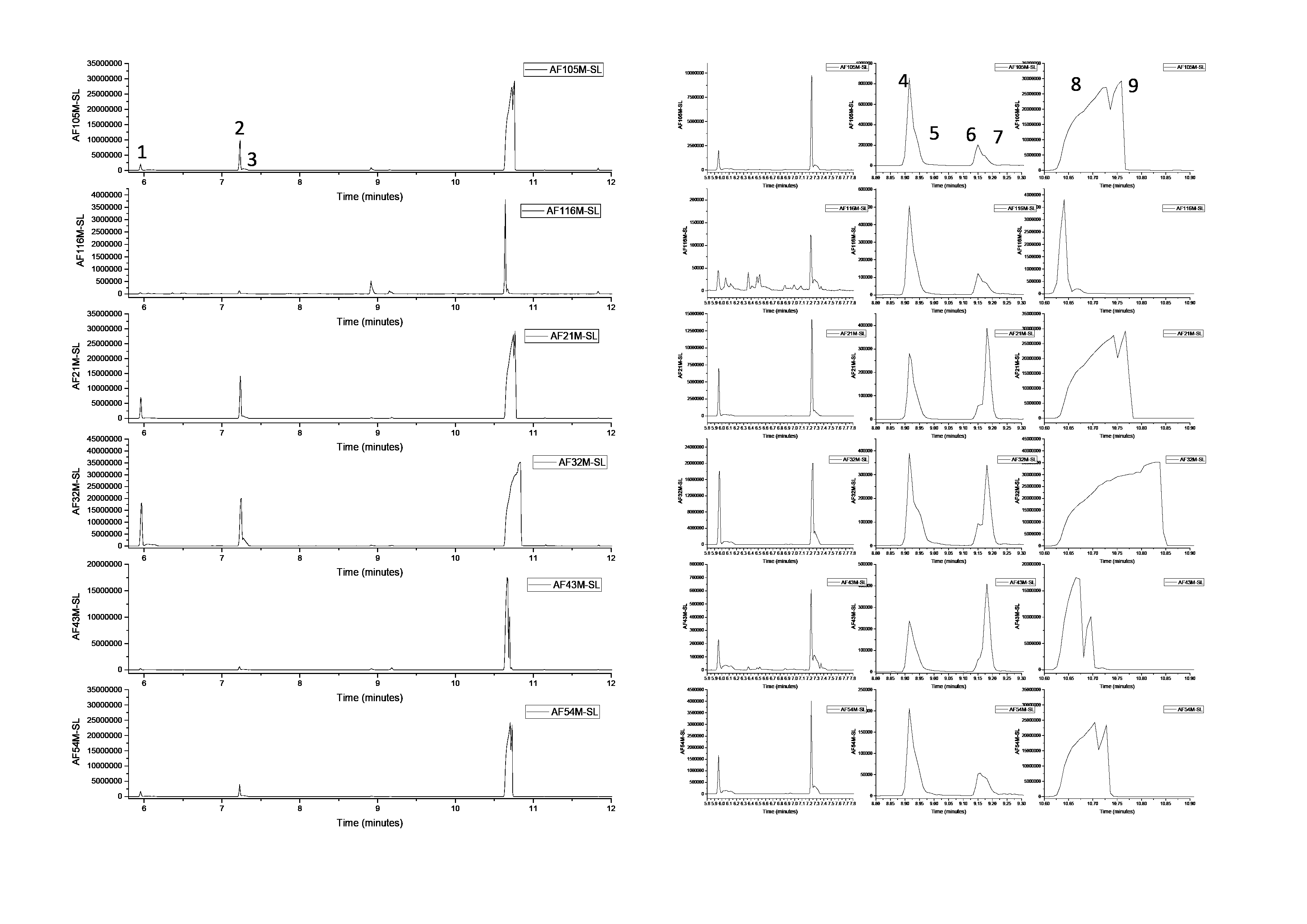

Supplement: Supplementary file 1 — Supplementary Material 1 [file 10886_2024_1508_MOESM1_ESM.png]

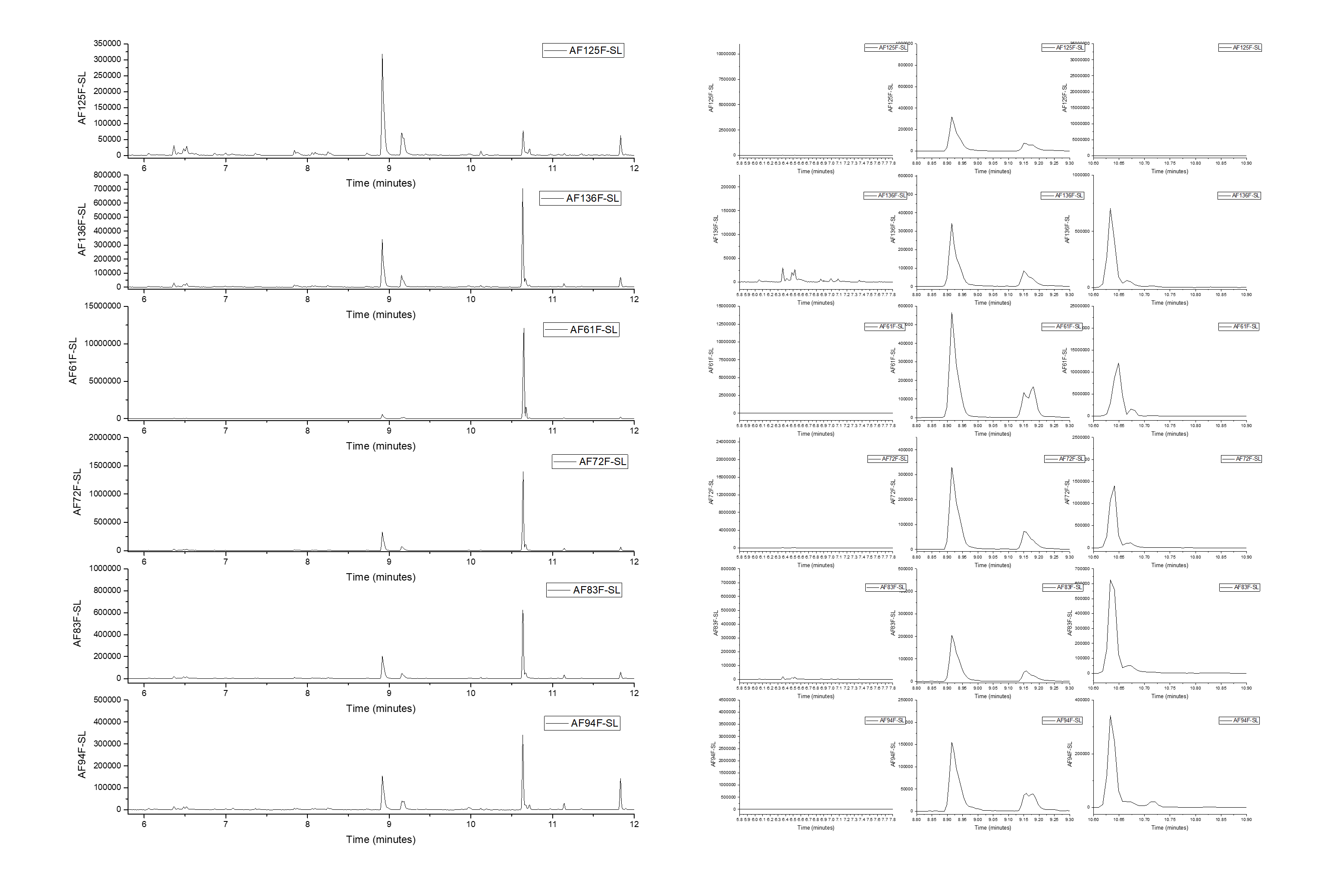

Supplement: Supplementary file 2 — Supplementary Material 2 [file 10886_2024_1508_MOESM2_ESM.png]

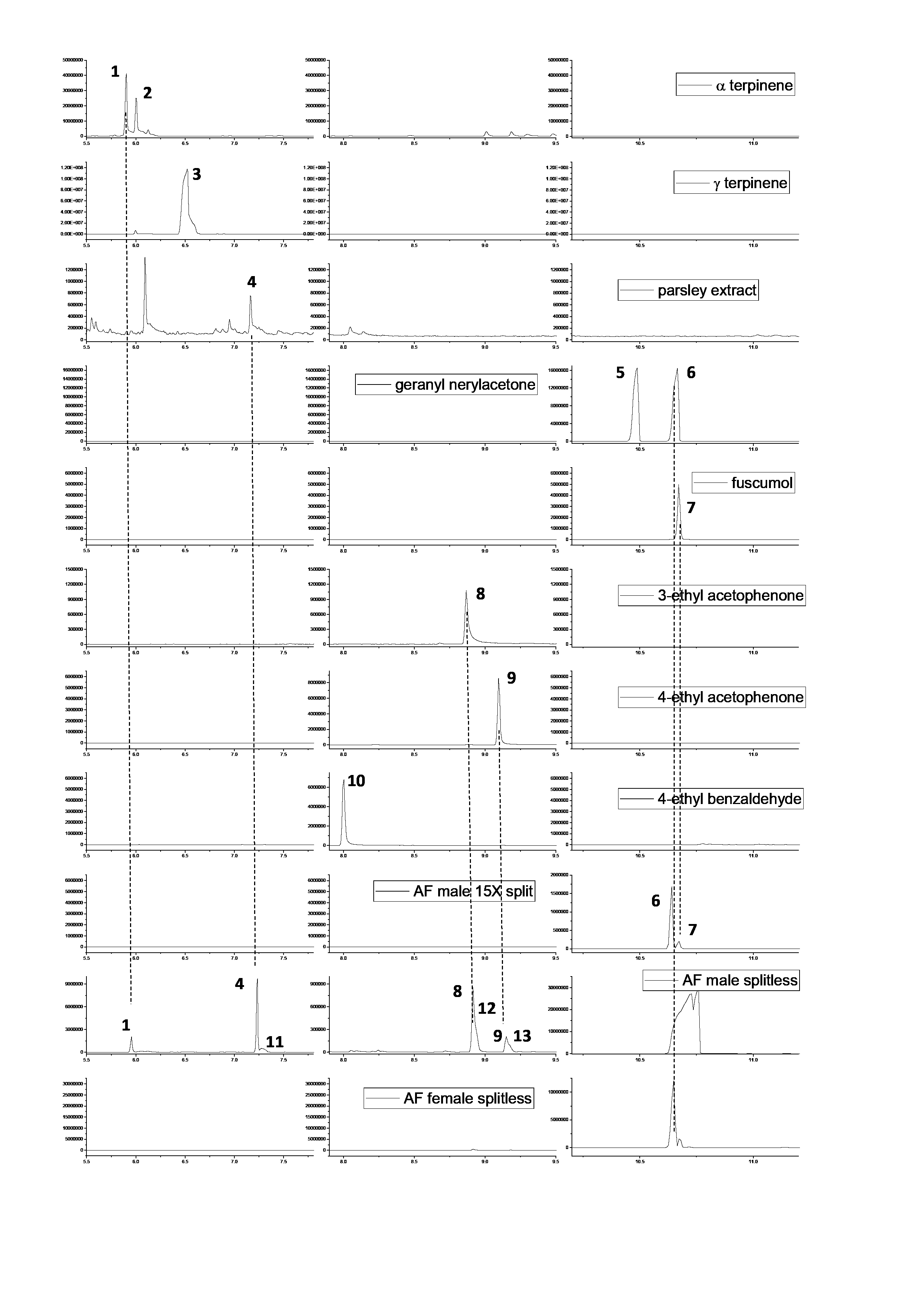

Supplement: Supplementary file 3 — Supplementary Material 3 [file 10886_2024_1508_MOESM3_ESM.png]

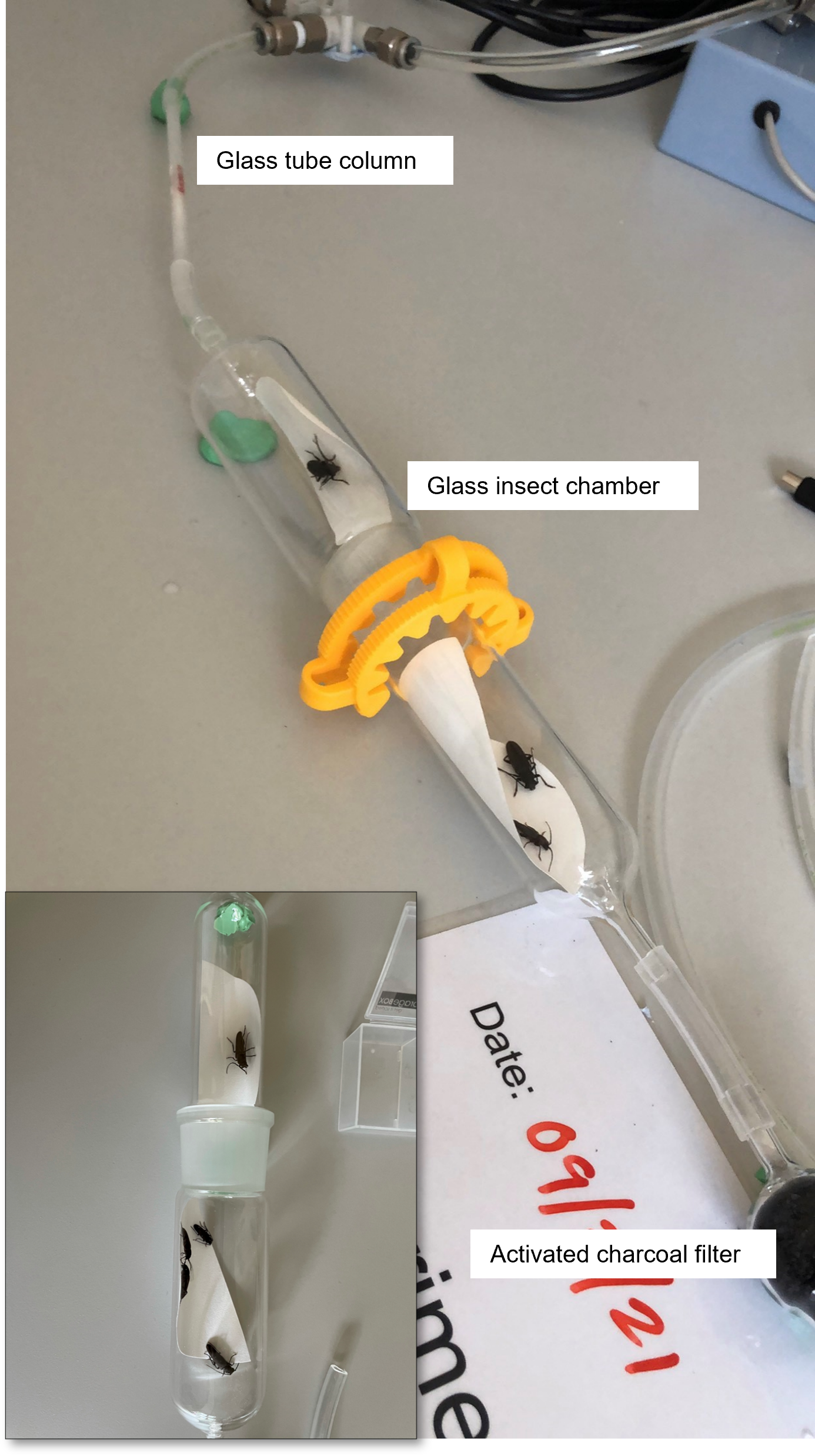

Supplement: Supplementary file 4 — Supplementary Material 4 [file 10886_2024_1508_MOESM4_ESM.png]
