# Supplementary material for: Exploring the Nature of Arhopalus ferus (Coleoptera: Cerambycidae: Spondylidinae) Pheromone Attraction
Source: J Chem Ecol. 2024 Jun 6;50(12):904–19. doi: 10.1007/s10886-024-01508-8 (PMC11717888; doi:10.1007/s10886-024-01508-8)
Supplement: Supplementary file 5 — Supplementary Material 5 [file 10886_2024_1508_MOESM5_ESM.docx]

**Supplementary Table S1. Percent purity, load per septa, and average release rate at 21°C from rubber septa loaded with fuscumol, fuscumol acetate, geranylacetone or GC-MS glass vials with** α-terpinene, **used to test attraction of burnt pine longhorn beetles, *Arhopalus ferus*, in New Zealand forests over three field seasons.**

| **Compound** | **% Purity** | **Load in**  **septum or glass vial (mg)** | **Mean release rate (mg/d) at 21°C** |
| --- | --- | --- | --- |
| **Racemic *E/Z*-fuscumol** | **99%** | **50** | **0.7** |
| **Racemic *E-*fuscumol** | **95%** | **50** | **0.7** |
| ***S-E-*fuscumol** | **94%** | **50** | **0.7** |
| ***R-E-*fuscumol** | **75%** | **50** | **0.7** |
| **Racemic *E/Z*-fuscumol acetate** | **95%** | **50** | **0.5** |
| ***E/Z-*geranylacetone** | **95%** | **78** | **2.1** |
| ***E*-geranylacetone** | **95%** | **78** | **2.1** |
| *α*-terpinene | **95%** | **1000** | **3.04** |

**Supplementary Table S2. Results of Sidak post-hoc contrasts comparing mean log transformed, normalized electroantennogram (EAG) responses (log(mV)) of antennae excised from adults of the burnt pine longhorn beetle, *Arhopalus ferus,* to different enantiomers of fuscumol (i.e.,** (*R*)*-*(*E*)-fuscumol, (*S*)*-*(*E*)-fuscumol, and racemic (*E,Z*)-fuscumol) applied at doses of 0.1, 1, 10 and 100 ug. Antennal responses were compared between compounds when applied at the same dose. For each comparison, the estimate is the average difference in response to the respective compounds; *P* values in bold font indicate statistically significance (α = 0.05)

| **Comparisons** | **Dose** | **Estimate** | **SE** | **df** | ***T*** | ***P*** |
| --- | --- | --- | --- | --- | --- | --- |
| **(*R* )-fuscumol vs racemic fuscumol** | **0.1** | **-0.38** | **0.22** | **81.7** | **-1.66** | **0.27** |
| **(*R*)-fuscumol vs (*S*) -fuscumol** | **0.1** | **0.02** | **0.22** | **88.9** | **0.09** | **0.99** |
| **Racemic fuscumol vs (*S*)-fuscumol** | **0.1** | **0.40** | **0.22** | **88.9** | **1.79** | **0.21** |
| **(*R*)-fuscumol vs racemic fuscumol** | **1** | **-0.65** | **0.23** | **81.7** | **-2.86** | **0.016** |
| **(*R)*-fuscumol vs (*S*)*-*fuscumol** | **1** | **-0.91** | **0.22** | **88.9** | **-4.07** | **0.0003** |
| **Racemic fuscumol vs (*S*)-fuscumol** | **1** | **-0.35** | **0.22** | **88.9** | **-1.14** | **0.60** |
| **(*R*)-fuscumol vs racemic fuscumol** | **10** | **-0.77** | **0.23** | **81.7** | **-3.40** | **0.003** |
| **(*R)*-fuscumol vs (*S*)*-*fuscumol** | **10** | **-0.53** | **0.22** | **88.9** | **-2.36** | **0.06** |
| **Racemic fuscumol vs (*S*)-fuscumol** | **10** | **0.25** | **0.22** | **88.9** | **1.11** | **0.61** |
| **(*R*)-fuscumol vs racemic fuscumol** | **100** | **0.07** | **0.27** | **85.5** | **0.27** | **0.99** |
| **(*R)*-fuscumol vs (*S*)*-*fuscumol** | **100** | **-0.12** | **0.26** | **95.0** | **-0.45** | **0.96** |
| **Racemic fuscumol vs (*S*)-fuscumol** | **100** | **-0.19** | **0.26** | **90.5** | **-0.75** | **0.84** |
