# Supplementary material for: Exploring the Nature of Arhopalus ferus (Coleoptera: Cerambycidae: Spondylidinae) Pheromone Attraction
Source: J Chem Ecol. 2024 Jun 6;50(12):904–19. doi: 10.1007/s10886-024-01508-8 (PMC11717888; doi:10.1007/s10886-024-01508-8)
Supplement: Supplementary file 6 — Supplementary Material 6 [file 10886_2024_1508_MOESM6_ESM.docx]

**Supplementary Fig. S1.** Image showing equipment used for 24-h volatile collections from *Arhopalus ferus* males and females, indicating glass insect chamber, glass tube columns, and activated charcoal filter. Insert shows a close-up of insects in the glass chamber resting on filter paper.

**Supplementary Fig. S2.** Gas chromatography traces of known standards (alpha terpinene, fuscumol, geranyl nerylacetone) showing the compounds with retention times matching those of compounds observed in GC traces of 24-h collections of volatiles from *Arhopalus ferus* males and females (bottom three rows). The y-axes represent total ion count (note the varying scales) and the x axis is time (min). Each trace (row) has been divided into three sections to allow the y-axis scale to vary and more clearly show retention time of compounds present in lower concentrations. The major components were geranylacetone (6) and fuscumol (7) observed in males and in much smaller amounts in females; minor components were α-terpinene (1) and *p*-mentha-1,3,8-triene (4) observed in males only. Traces of 3-ethyl acetophenone (8), 4-ethyl acetophenone (9), unknown (11), unknown (12), and siloxane (13) were observed in male samples as well as some control samples. Compounds (peaks) observed in injected standards but not in *A. ferus* samples were para cymene (2) – a degradation product of α-terpinene, gamma terpinene (3), and 4-benzylaldehyde (10).

**Supplementary Fig. S3.** Gas chromatography-mass spectrometry (GC-MS) analyses of 24-h collections of volatiles from five different groups of *Arhopalus ferus* males. Each group consisted of five males. Left graph stack, whole GC-MS trace, Right graph stack, magnified in timeframes of relevance. Showing TIC, total ion current on y-axis. Major components were geranylacetone (8) and fuscumol (9) observed in males and in much smaller amounts in females; minor components were α-terpinene (1) and *p*-mentha-1,3,8-triene (2) observed in males only. Traces of 3-ethyl acetophenone (4), 4-ethyl acetophenone (6), *p*-mentha-1,5,8-triene (3), unknown (5), and siloxane (7) were observed in male samples as well as some control samples.

**Supplementary Fig. S4.** Gas chromatography-mass spectrometry (GC-MS) analyses of 24-h collections of volatiles from five different groups of *Arhopalus ferus* females. Each group consisted of five females. Left graph stack, whole GC-MS trace, Right graph stack, magnified in timeframes of relevance. Showing TIC, total ion current on y-axis.
